# Current state and call for action to accomplish findability, accessibility, interoperability, and reusability of low carbon energy data

**DOI:** 10.1038/s41598-022-08774-0

**Published:** 2022-03-25

**Authors:** Valeria Jana Schwanitz, August Wierling, Mehmet Efe Biresselioglu, Massimo Celino, Muhittin Hakan Demir, Maria Bałazińska, Mariusz Kruczek, Manfred Paier, Demet Suna

**Affiliations:** 1 Western Norway University of Applied Sciences, Department of Environmental Sciences, Sogndal, 6856 Norway; 2 Izmir University of Economics, Sustainable Energy Division, Izmir, 35330 Turkey; 3 ENEA, Lungotevere Thaon di Revel, 76, 00196 Roma, Italia; 4 Central Mining Institute, Katowice, 40-166 Poland; 5 AIT Austrian Institute of Technology, Vienna, 1210 Austria

**Keywords:** Energy science and technology, Climate sciences

## Abstract

With the continued digitization of the energy sector, the problem of sunken scholarly data investments and forgone opportunities of harvesting existing data is exacerbating. It compounds the problem that the reproduction of knowledge is incomplete, impeding the transparency of science-based targets for the choices made in the energy transition. The FAIR data guiding principles are widely acknowledged as a way forward, but their operationalization is yet to be agreed upon within different research domains. We comprehensively test FAIR data practices in the low carbon energy research domain. 80 databases representative for data needed to support the low carbon energy transition are screened. Automated and manual tests are used to document the state-of-the art and provide insights on bottlenecks from the human and machine perspectives. We propose action items for overcoming the problem with FAIR energy data and suggest how to prioritize activities.

## Introduction

In a seminal publication, Wilkinson et al.^1^ formulated the so-called FAIR principles to promote the sharing of data, scientific data management, and stewardship. FAIR stands for findability, accessibility, interoperability, and reusability of data. Our study is a response from the low carbon energy research domain to a call for action in this paper. Therein, the authors urge ’all data producers and publishers to examine and implement ... (the FAIR) principles and actively participate with the FAIR initiative ...’. We respond to this call by documenting the state-of-the-art on FAIR data practices in our domain and suggest action items, drawing from an examination of 80 databases representative for data flows necessary for the low carbon energy transition. Our assessment follows the recommendations by Wilkinson et al.^2^, see ’features that should be reflected’.

**Figure 1:**
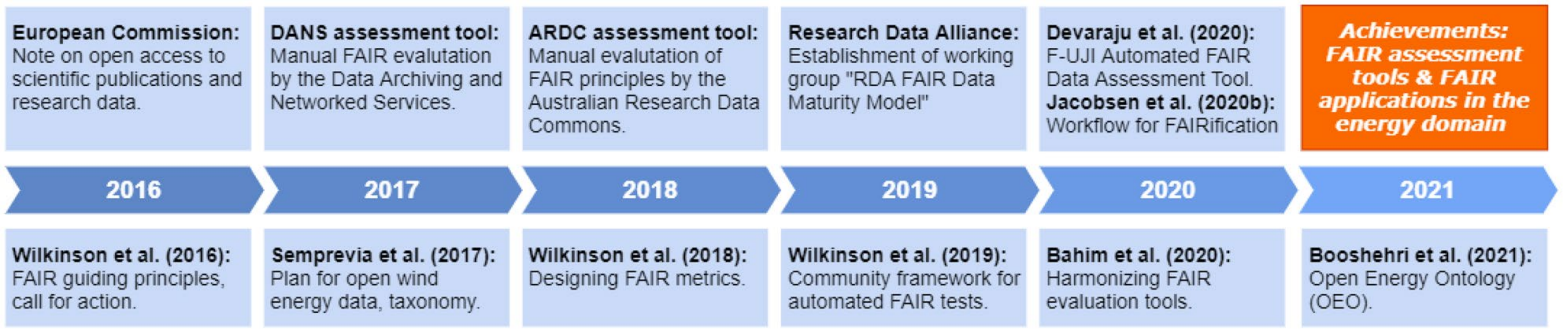
Selected milestones toward the implementation and testing of FAIR guiding principles along with efforts undertaken in the energy domain.

The FAIR Principles^1^ have widely been acknowledged as the way forward for improving the findability, accessibility, interoperability and reusability of data across different sources and disciplines^3–5^. Various research communities are currently discussing and testing how to implement these guiding principles^6–11^. Figure 1 presents selected milestones toward the testing and implementing of the FAIR guiding principles and efforts undertaken in the energy domain. Currently, very few initiatives exist among low carbon energy researchers to progress the state-of-the-art^12–14^. A key element of the implementation of FAIR data principles is the introduction of standard metadata information and its proper referencing. Sempreviva et al. (2017)^15^ pioneered the development for wind energy research by suggesting a taxonomy and metadata. This taxonomy intends to support open data access, using a cloud-based data portal. Booshehri et al.^13^ introduced the Open Energy Ontology (OEO), which compiles standards for defining energy technology concepts and related infrastructure (e.g., the concept of district heating and its implementation by cogeneration power plants). Independent of a specific domain, several evaluation tools have been developed to test the compliance of data resources with the FAIR principles and to offer guidance in improving the FAIR status. These tools range from checklists^16–18^ and ^19,20^ to (semi-)automated evaluators^2,21–24^.

**Figure 2:**
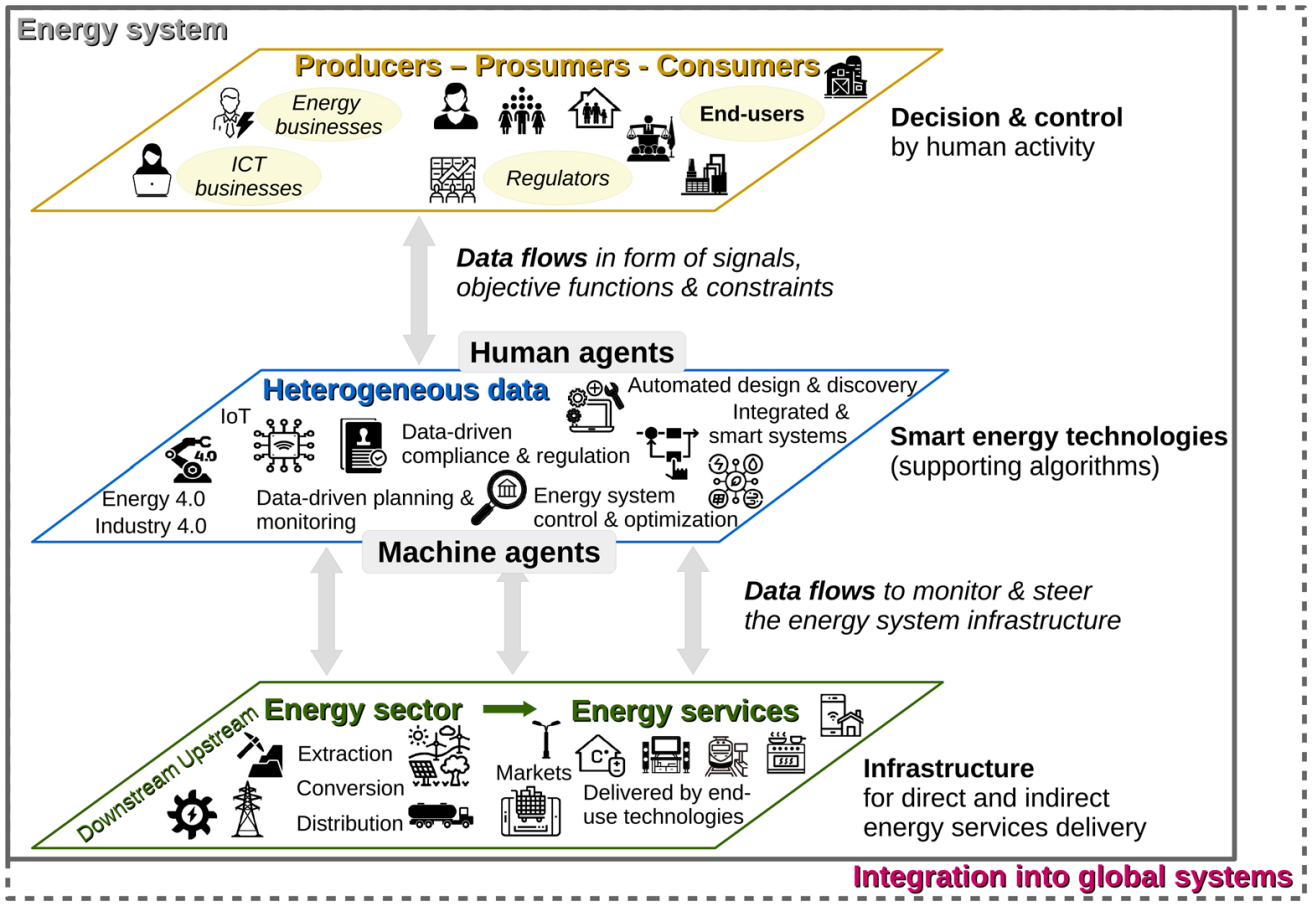
The energy system with human and machine agents at the center. The top layer details human actors in the energy sector, engaged in the production, distribution, and/or consumption of energy services. Their decisions and behaviors define the objectives and constraints of the energy system. This information is delivered through bilateral heterogeneous data bundles that are taken up by smart energy technologies to monitor and steer the energy system infrastructure (bottom layer).

In implementing the FAIR principles, energy science faces a triple challenge. First, it is crucial to meet the needs of a broad range of data stakeholders, who include researchers from social sciences to engineering, energy and other industries, policy- and decision-makers, funding and publishing agencies, and the general public. While domain experts need data at high granularity, other stakeholders require information at an aggregate level. Data needs also differ from stakeholder to stakeholder^12^. For example, utility companies rely on high-resolution electricity demand data, while policy planners are more interested in aggregated trends in different fields. Energy researchers utilize a broad range of data, covering technical specifications to societal and environmental impacts. Notably, data not only support knowledge building and validation, but also provide important input to decide on pathways for the transition to a low carbon energy system^25,26^. Second, data in the energy system cover large scales in time and space, respectively ranging from picoseconds to geological age (e.g., technical dispatch vs. the formation of energy resources) and nanoscale to the planning horizon of humanity (e.g., unit-level control of the electricity grid vs. long-term planning of secure access to pivotal resources in respect of planetary boundaries)^27,28^. Third, a new type of agent beyond humans emerges in the energy system: automatized decision and control systems (machines) support human activity in supervising the energy infrastructure. This, in turn, requires that data need to become machine-actionable. Machine-actionability means that machines can be programmed so that they find, access, and process data—ultimately without further human interaction. The implications of this third challenge lead to a new perspective on an energy system with human and machine agents at the center^15,29,30^. The new perspective on the energy system is visualized in Fig. 2.

Three layers (top, middle, and bottom) show how the energy system is controlled and steered by these two types of agents, enabling the provision of energy services in demand. Energy service refers to the direct and indirect use of energy for heating, lighting, air-conditioning, transportation of people and goods, long-distance communication, and the production of clothes, houses, cars, and food, etc.^31–35^. The entire energy system is service-driven, and processes such as energy generation and distribution are analyzed and planned in the context of what energy is used for^36^. The infrastructure for energy services delivery is more and more data-driven. Bidirectional flows of data provide the foundations for humans and machines to manage this infrastructure. The top layer, ’Decision & control by human activity’, depicts the decision and control activities in the energy system^37^. The classical boundaries between producers on the one hand and consumers on the other hand are thereby disappearing, making room for ’prosumers’^38^. The top layer also emphasizes the new, significant role of ICT businesses in addition to businesses involved in monitoring, operating, and managing energy service provision (energy businesses). The middle layer complements human control of the energy system with machine control by the help of ’smart energy technologies’. The support from machines to steer the energy infrastructure (bottom layer) depends on enabling technologies such as data-driven regulatory systems with feedback and adaptive behavior, infrastructure control, flagging of alerts, real-time monitoring, data-driven compliance, and regulation. An example is the tracking and monitoring of CO_2_ emissions linked to energy services provision. The importance of data is expected to grow further in the future with the continued digitization of the energy sector, in particular with the broad introduction of smart and AI-based technologies in support of decision-making and real-time system adaptation^39,40^. Moreover, algorithm-based strategies to identify technological solutions are increasing. An example is the automatized material selection and design without the need of expensive and/or risky experiments^41^. Consequently, market opportunities for sharing data are expected to grow tremendously, provided that the bottlenecks for doing so are removed.

The main task of machine agents is to support the infrastructure needed to deliver energy services (bottom layer). This includes the extraction and harvesting of energy resources, the conversion between different forms of energy to useful energy, the distribution of fuels, as well as the operation and maintenance of the energy equipment across temporal and spatial scales. Data streams flowing between the top and the middle layer are input to machines in the form of signals, objective functions, and constraints. These include taxes on energy fuels (e.g., connected with Greenhouse Gas emissions), R&D programs, energy security targets, health and sustainable development goals, as well as data security and privacy requirements. The bottom layer exchanges data with smart energy technologies to provide the foundations for humans and machines to manage the necessary energy infrastructure.

Given the above rationales to support the sharing of energy data between layers of the energy system, the task ahead for the energy research community is to find a domain-specific way forward to implement the FAIR data guiding principles. The way forward depends on a consensus in the community about the prospects of FAIR data for low carbon energy research. This includes, on the one hand, clarity about connected costs (time, person months), and on the other hand, a shared understanding about the economic and social value that lies in FAIR data. The collective recognition is a prerequisite for functional FAIR data markets and the establishment of supporting mechanisms. How to do this is an open question to researchers and practitioners alike. As a first step, teams need to evaluate the costs and benefits for FAIRifying their data sets, ’allowing members of that community to evolve over time while realistically operating within their budgets in order to achieve their best FAIR performance’^2^. It starts with the recording of the status quo to spark discussions. Crucial for such a process is the evaluation of the current state of implementation of the FAIR principles in the energy domain. This communication aims at advancing such an evaluation with the help of evaluation tools developed by various initiatives. In line with Wilkinson et al.^2^, the point of the evaluation of the current level of FAIR implementation in the energy domain is to identify ’opportunities for improvements’ instead of seeing scores as a goal in themselves. Figure 2 serves as the starting point for the assessment. With its help, we choose a representative sample of 80 energy databases that cover the current and emerging energy system and connected data flows relevant for the low carbon energy transition. To test whether the choice of databases is representative of data flows in the energy system, we reflect the Global Energy Assessment Report^42^ in Fig.2. We also use established classification schemes for energy data to complete the picture. We do so by mapping tested databases onto key tasks for enabling the energy transition. Refer to the Method Section below and the Supplementary Material for details. For the following two reasons, we restrict ourselves to testing databases relevant for understanding the low carbon energy transition. First, the energy system is in a transition to a system dominated by low carbon technologies, and fossil fuel based technologies will be phased out. Second, the implementation of FAIR principles advances the transparency of transformation of the energy system and, thus, supports the public acceptance of the transition processes.

## Results

**Figure 3:**
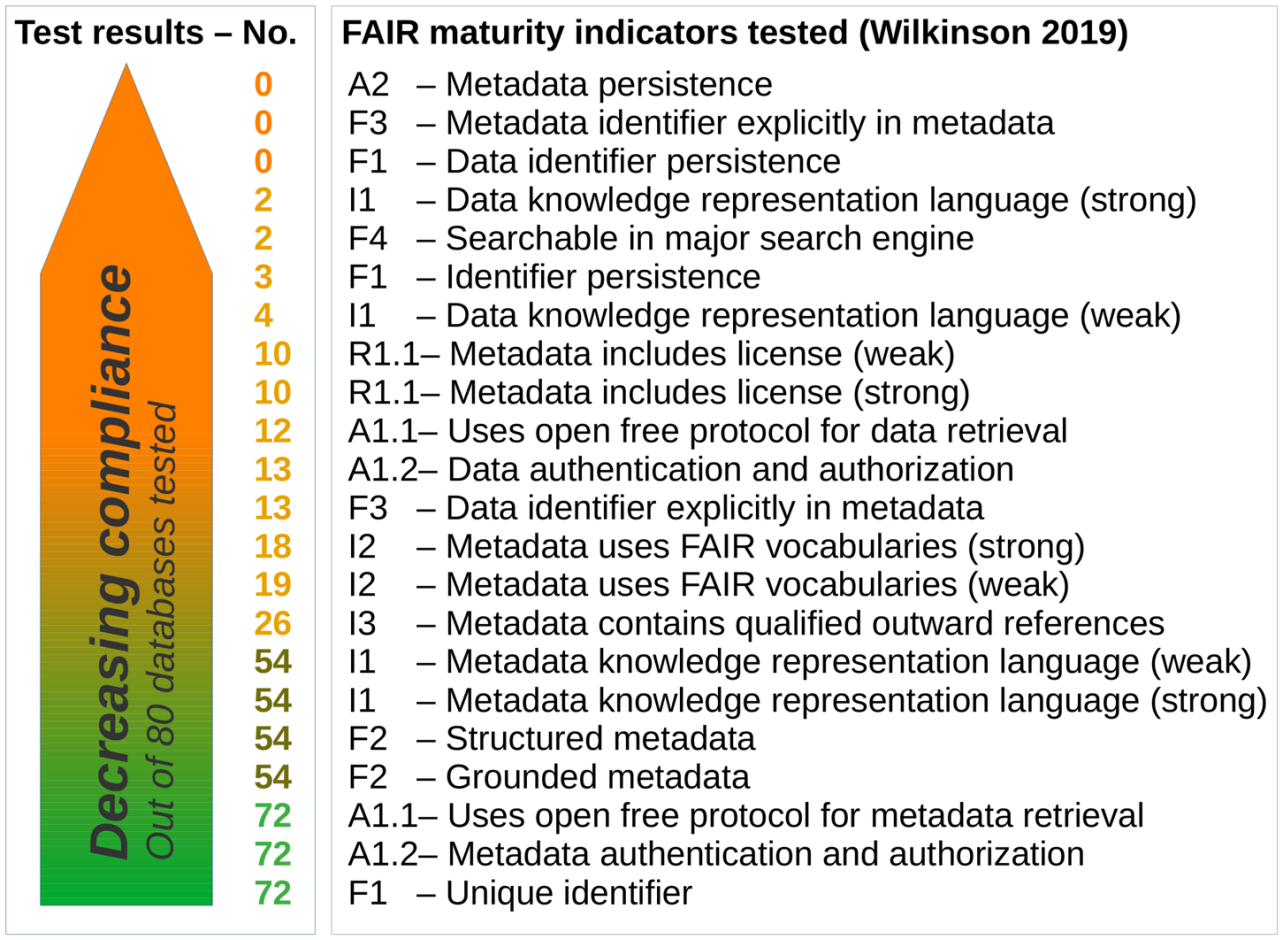
Number of databases complying with FAIR maturity indicators as operationalized in Wilkinson et al.^2,43^ that test 13 of 15 FAIR principles. The results are based on machine-actionability tests for 80 databases that are representative of data flows for low carbon energy research. None of the tested databases achieves persistence of metadata and data identifiers. Internal linking of metadata with the help of identifiers is equally problematic.

We assess a corpus of 80 databases that is representative of data flows that are pivotal for the low carbon energy transition. The selection of these databases is guided by Fig. 2. In addition, an ontology developed from the Global Energy Assessment Report^42^ was used. The proof of representativeness is described in the Supplementary Material. We assess the compliance of the selected databases with the FAIR guiding principles using an automated assessment tool^2^. Figure 2 summarizes the results, sorting the compliance of databases with the 22 FAIR maturity indicators implemented by Wilkinson in the ’FAIR maturity evaluation service’^2^. Quoting the information from the website^44^, the maturity indicator ’authentication and authorization of metadata’, e.g., tests ’metadata GUID (global unique identifier) for the ability to implement authentication and authorization in its resolution protocol’, whereas the indicator ’data identifier persistence’ reports on a ’metric to test if the unique identifier of the data resource is likely to be persistent’.

We find that most of the energy databases allow the authentication and authorization of metadata, use open free protocol for metadata retrieval, and incorporate unique identifiers. 72 out of 80 databases are compliant, see bottom of Fig. 2. At the same time, none of the tested databases achieves persistence of metadata and data identifiers (top of Fig. 2). A general observation is also that the majority of databases poorly comply with the FAIR maturity indicators. This echoes findings from a similar screening of databases performed by the project EOSC-Nordic^45^. Contrary to our study, this analysis has a geography focus on repositories from Nordic countries and is not domain-specific.

Two thirds of the databases do not fulfill 15 out of 22 indicators. This highlights the urgency to improve the FAIR state of energy (meta)-data and stresses the general lack of machine-actionability. Without machine-actionability, opportunities in harvesting data for society will not materialize.

**Figure 4:**
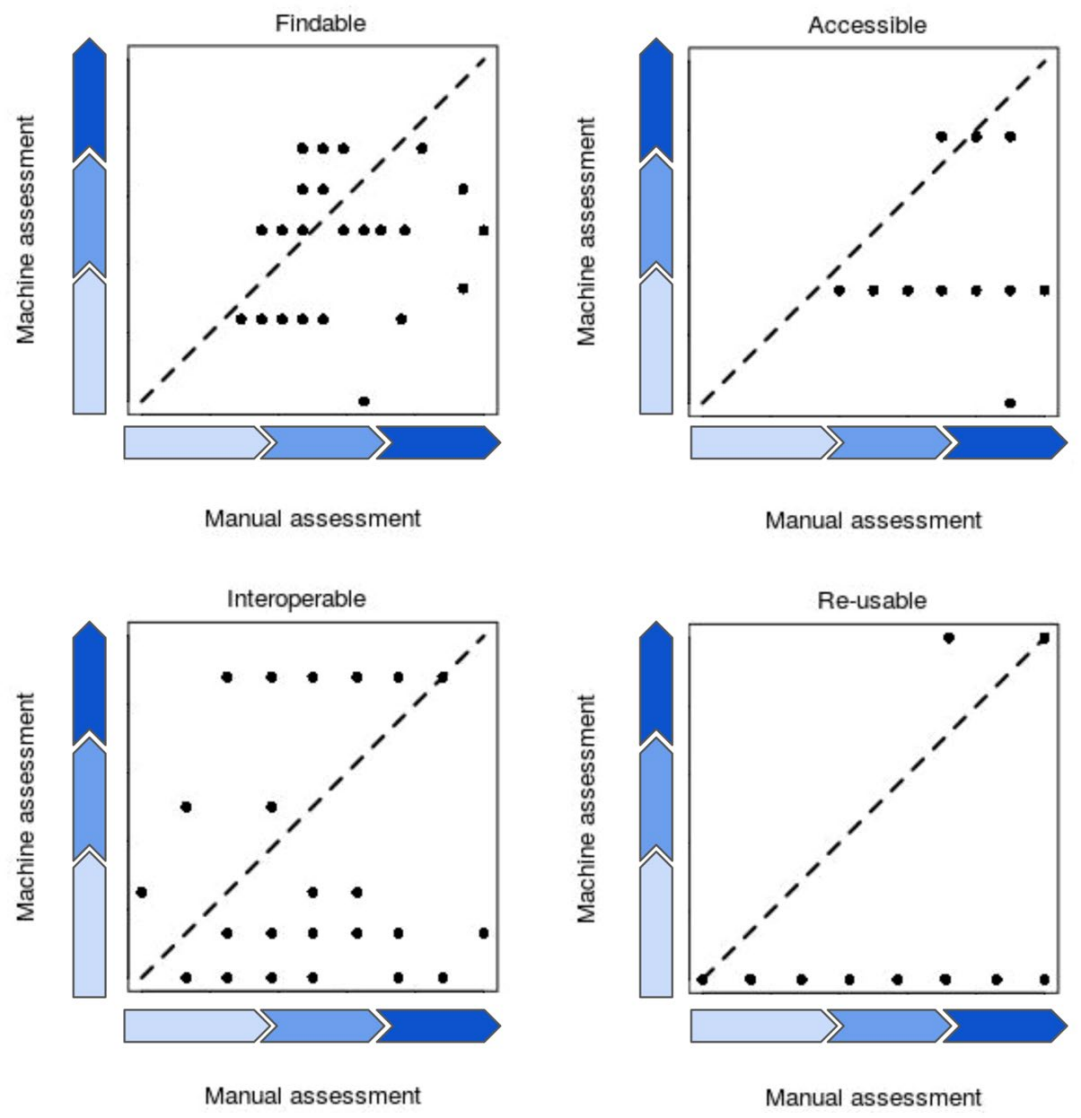
Stylized comparison of manual vs. machine assessments.

In addition to machine assessments, 30 of the 80 databases were also evaluated manually using the ARDC self-assessment tool^46^, among others. As documented in the Supplementary Material, we detect a large spread of results in the manual assessments of the same databases by different researchers. The largest spread was found for the assessment of the interoperability of data. This underscores, first, a strong degree of subjectivity in the assessments, originating from the different disciplinary background and data governance proficiency of the analyst. Secondly, a shared understanding about what makes data FAIR is lacking. Furthermore, the comparison of automated and manual assessments allows us to contrast the machine and human perspectives on FAIR evaluations in the energy domain. To this end, the original weighing of answers to the ARDC assessment questions has been transferred to the machine-actionable FAIR maturity test (refer to the Supplementary Material for the details). Figure 3 shows the stylized results of this comparison for each of the FAIR guiding principles (findability, accessibility, interoperability, and reusability). We abstain from reporting assessment scores to focus on observed gaps and the room for improvement (see Wilkinson et al. 2019^2^).

A tendency is that machine assessments score lower than manual assessments with the exception of the interoperability criteria where the results are mixed. This tendency has also been observed in a recent FAIR assessment of databases from the World Data Center for Climate^47^. A reason for the overall lower scoring by machines is that the assessment is strictly binary—either the test is fully compliant or not at all. In contrast, the manual assessment allows for nuances, but they are subject to interpretation by the user. We also find that metadata do not point to and identify the data they are describing. Most websites are designed to solely cater to a human data selection process. Moreover, many providers of data offer interfaces to data and not the data themselves. In these cases, the design is not suitable for machine access. Examples include drop-down menus or hover boxes for value selection. Accessibility to (meta-)data is mostly impeded because metadata are not persistent. Among the bottlenecks for Findability are missing metadata pointers, long-term and stable access to data, and searchability of (meta-)data. At the same time, we do not observe lower scores for both machine and manual assessments. The same observation holds for the Accessibility criteria. The simple reason behind it is a selection bias—we study databases that are findable and accessible—at least through a website. This would change if a scalable, automated test, as suggested by Wilkinson et al. 2019^2^, existed (or even crawling websites to find assessment candidates). Reusability is an issue because machines do not find license information, even if available for humans (hence the binary scoring results for machines). For Interoperability to work, data would need a much better standardized description of what they are about. A good example is the approach proposed for the smart grid by Huang et al. 2017^48^. We also find that Interoperability is assessed most differently from the human and machine perspective (Fig. 3). A lack observed from a human point of view is that only islands of standardized knowledge representation and terminology exist; even less often are they interlinked, which would allow for the navigation of data and metadata from one field of expertise to the next. From the machine perspective, a standardization of vocabularies along with the pointers to its place of definition is indispensable. For example, while ”Kilowatt hour” bears a meaning to users of energy data, machines need a semantic definition as, e.g., provided by QUDT^49^, an organization promoting the interoperability of data and the specification of information structures through industry standards for units of measure, quantity kinds, dimensions, and data types. In this case, ’unit:KiloW-HR’ is defined via the uniform resource identifier ’http://qudt.org/vocab/unit/KiloW-HR’. The problem is that semantic definitions are still scarce, little known, and even less often implemented.

Finally, we report that energy databases differ greatly in content, size, layout, and formats. Databases can store instrument readings such as metering data, data on price developments in the energy markets, and material composition of the electricity grid. This heterogeneity of energy data presents a grand challenge for scaling up database assessments. In sub-domains such as energy statistics, standards already exist^50^. Also, information exchange about electric power system components is regulated by the Common Information Model^51^, a standard set by the International Electrotechnical Commission, to allow standardized interfaces for software applications. A lot can be gained by, e.g., agreeing on common data formats as well as having well-defined, machine-actionable references to fundamental concepts and terms used in the energy sector. However, for the energy system as a whole, the current routines and tools are not up to the task, making the energy domain an excellent test-bed for improvements in this direction.

## Discussion

The results disclose the difficulty of translating the FAIR guidance principles into domain-specific applications, as current FAIR data practices in the energy domain are still in its infancy. Although the low carbon energy community has started efforts of FAIRifying energy data, platforms and tools are not yet fit to be integrated into the workflows of research teams. Most importantly, machine-actionability is not given at large.

This study is the first to assess and document FAIR data practices in the energy domain. We test 80 databases that are representative of data flows in the energy system with the help of manual and machine-based assessments. The comparison offers several novel insights, suggesting how to move forward in and with the community. We recommend the following action items for the energy domain (in order of priority):

1. Create institutions or networks that can function beyond single project and which are responsible for defining domain-specific, machine-actionable standards. Institutional anchoring of metadata and domain-specific vocabulary can increase trust and confidence into the uptake of the work of pioneers and investments into FAIR work-flows. Another task for these institutions or networks could be to coordinate the future energy data space^12,52^. This data space should serve as an entry point to FAIR data tools, workflows, and semantic web-services specific to the energy domain, besides ensuring interoperability with data spaces of other domains.
2. Our findings show that both manual, as well as automated FAIR assessment tools, lead to divergent recommendations on improving the FAIR status of databases. In order to provide the community with meaningful tools and to foster confidence in the outcome of the evaluation results, it is necessary to harmonize the tools. The FAIR Data Maturity Model put forward by the Research Data Alliance is a step forward in this direction^53^.
3. Approach the overall lack in understanding of how to implement machine-actionability through demonstrated use cases in the energy domain. Using a simple structured dataset, a blueprint can be developed to show how to enable machine-actionability. The use case illustrates how to assign persistent identifiers to (meta)data, link to existing standards, and assign licenses and access rights. The encouragement of peer-reviewed publications of such blueprints also addresses the incentive problem for investing into FAIR research data.
4. Harvest low-hanging fruits by placing emphasis on the implementation of persistent identifiers for (meta-)data. Several repositories are offering these services.
5. Promoting and educating FAIR energy data stewards. The technical expertise and the resources needed to FAIRify energy data is out of reach for energy researchers. Even if assessment tools are available to support self-assessment of research data, the cycle of developing, assessing, and improving data documentation is out of scope for daily activities. In particular, the task of FAIRifying data connected to research publications should not be outsourced to the researchers.
6. Reverse the trend to prioritize the development of (graphical) user interfaces that prohibit the access to raw data. The assessments revealed that these interfaces are designed for human users only and are hardly machine-actionable.

## Methods

Assessing the state of FAIRness across low carbon energy research data relies on two basic steps: I) to define a corpus of relevant data and II) to apply a FAIR evaluation methodology to this body. While the methodology can be developed independently of the energy domain to some extent, the systematic and comprehensive compilation of the data corpus is a domain-specific task. Using the corpus of relevant energy databases, assessment tools were applied to understand the overall compliance with the FAIR guiding principles, as well as issues concerning each of the four principles. In our assessment of energy databases, we follow the 9 features of the community-driven approach as suggested in Wilkinson et al.^2^: We carry out a number of assessment approaches, including automated assessments, assessments informed by the crowd (by drawing from a series of discussions about databases and FAIR gaps observed in the energy community, see Wierling et al. 2021^12^), and through intensive discussions within the group of authors of this publication. We also use a range of tools to assess the same databases. Most importantly, we abstain from a fixation on the assessment cores, which is why we do not report any score in the final figures. Instead, we embrace the idea that ’an intrinsic value’ of scores is absent. Rather, assessment should be used as guidance to draw conclusions for the way forward in improving the status quo of FAIR implementation in the domain.

We compile and select 80 databases representative of data flows in the energy system (Fig. 1). We test how representative our choice is with the help of an ontological concept based on Fig. 1, reflecting the importance of data flows in the energy system. The ontology draws from the Global Energy Assessment Report^42^ and established classification schemes for energy data, such as the Standard International Energy Product Classification by UNSTATS and IRES^50^, the Global Change Master Directory Keywords^54^, JEL classification Codes^55^, and the European Science Vocabulary^56^. Table 3 in the Supplementary Material presents the 80 databases vis-a-vis key concepts. When choosing the databases, additional care has been taken to ensure a wide spread across hosts of databases (incl. general purpose repositories, institutional repositories—public and private, single databases, and data sets, incl. data sets published as supplementary material to scientific publications). The uptake of the FAIR principles has been rapid, leading at the same time to manifold interpretations and, consequently, various assessment frameworks and metrics. Certainly, it is a very active area of research. Given the aforementioned role of automated services in the future energy system, an assessment of the machine-actionability of databases is of particular interest. Naturally, this can be best tested with an algorithmic framework. Indeed, the plethora of FAIRness claims and assessment tools led to stating the FAIR principle more precisely on the one hand^57^, and the development of automated tools on the other hand^2,21,24,58^.

We review available FAIR data assessment tools for manual and machine use. A overview of existing tools is available at the website fairassist.org. We select the ARDC DAIR data self-assessment tool^46^ for manual assessment and the FAIR indicator maturity test^2,44^ as one of the two available machine-actionable tests. The other one is the rapidly evolving F-UJI test^21^. A recently published third semi-automated assessment tool^24^ is specific for life sciences and therefore not considered in our context. The rationale for our choice of the ARDC FAIR data assessment tool is that it aligns with the FAIR principles and has a good balance between technical and non-technical questions (22 questions). The test allows scoring comparable to the machine test (12 tests) and was the only one available when this study was started. Although both tools have their own set of test questions, a mapping between them is possible at the level of each of the FAIR principles. The Supplementary Material details the approach and connected scores (Table 5).

30 assessments were carried out manually, while 80 tests are machine-based. The rationale for this is that we were already able to identify systematic patterns and adding more examples would not have led to a different picture. The number of assessed databases has been decided with the emergence of generalizable patterns for the state-of-the-art. Note also that manual assessment was carried out before and after a briefing on how to assess, with the intention of detecting the amount of subjectivity of tests. Figure 4 shows the example for the interoperability criteria, for others see the Supplementary Material.

The Supplementary Material provides further methodological details (Section 2), results from manual tests (Section 3.2), results from machine tests (Section 3.3, spreadsheet) and aggregate scores for the comparison (Section 3.1).

## Supplementary Information

Supplementary Information 1.

Supplementary Information 2.

## Data Availability

Supplementary Material—Machine tests (spreadsheet) https://doi.org/10.5281/zenodo.5577964. Supplementary Material—detailed description of the adopted methodology, results of manual and machine tests (Text document) https://doi.org/10.5281/zenodo.5578111.

## Code availability

There is no code associated with this study.
