# Supplementary material for: Current state and call for action to accomplish findability, accessibility, interoperability, and reusability of low carbon energy data
Source: Sci Rep. 2022 Mar 25;12:5208. doi: 10.1038/s41598-022-08774-0 (PMC8956656; doi:10.1038/s41598-022-08774-0)
Supplement: Supplementary file 2 — Supplementary Information 2. [file 41598_2022_8774_MOESM2_ESM.pdf]

Supplementary Material to the manuscript "Current state and call for action to accomplish findability, accessibility, interoperability, and reusability of low carbon energy data"

Authors: *Valeria Jana Schwanitz (Western Norway University of Applied Sciences), August Wierling (Western Norway University of Applied Sciences), Maria Bałazińska (Central Mining Institute), Mariusz Kruczek (Central Mining Institute), Demet Suna (AIT Austrian Institute of Technology), Christopher Burger-Scheidlin (AIT Austrian Institute of Technology), Manfred Paier (AIT Austrian Institute of Technology), Mehmet Efe Biresselioglu (IUE Izmir University of Economics), Muhittin Hakan Demir (IUE Izmir University of Economics), Massimo Celino (ENEA, IT)*

Content:

1. Proof of representativeness
2. FAIR/O assessment methods
    2.1 Comparing methods for FAIR assessments
    2.2 FAIR assessment tool by ARDC
    2.3 Machine-based assessment of FAIR criteria
3. Results
    3.1 Aggregated results human vs. machine assessments
    3.2 Results from manual assessments
    3.3 Results from machine-based assessments
4. References

1. Proof of representativeness

Data is omnipresent in the energy system, flowing between its agents supported by IT infrastructure (Fig. 1, main text). We assess the compliance of energy databases with FAIR and openness criteria. To test whether the choice of databases is representative of data flows in the energy system, we reflect Fig. 1 with the help of the Global Energy Assessment Report, GEA 2012, [1] and established classification schemes for energy data. We do so by mapping tested databases onto key tasks for enabling the energy transition. We utilize GEA 2012 as a reference framework, because it is a comprehensive review of the energy system drafted by numerous energy researchers from a broad range of disciplines. Altogether, 334 authors and 179 reviewers (including 5 anonymous reviewers) compiled a total of 1884 pages. This report on the energy system from 2012 is aiming to *"… examine: the major global challenges and their linkages to energy, the technologies and resources available for providing energy services, future energy systems that address the major challenges, the policies and other measures to realize sustainable energy futures."* Sustainable energy is defined by the three 'aspirational goals': "(1) *Stabilizing global climate change to 2 degrees above preindustrial levels to be achieved in the 21st century. (2) Enhance energy security by diversification and resilience of energy supply. (3) Eliminating household and ambient air pollution and universal access to modern energy services by 2030."* Box 1 below lists the chapters of GEA 2012. They illustrate the multi-disciplinary approach for examining the state of the art of the energy system and the sustainability challenges that lie ahead. Testing chapter by chapter (and sections), whether aspects covered in GEA 2012 are included in the energy system as presented in Fig. 1 (main paper), we find that this reference framework is well mirrored.

Box 1: Chapters of Global Energy Assessment Report, GEA 2012.

Chapter 1: Energy Primer
Chapter 2: Energy, Poverty and Development
Chapter 3: Energy and Environment
Chapter 4: Energy and Health
Chapter 5: Energy and Security
Chapter 6: Energy and Economy
Chapter 7: Energy Resources and Potentials
Chapter 8: Energy End-Use: Industry
Chapter 9: Energy End-Use: Transport
Chapter 10: Energy End-Use: Buildings
Chapter 11: Renewable Energy
Chapter 12: Fossil Energy
Chapter 17: Energy Pathways for Sustainable Development
Chapter 18: Urban Energy Systems
Chapter 19: Energy Access for Development
Chapter 20: Land and Water: Linkages to Bioenergy
Chapter 21: Lifestyles, Well-Being and Energy
Chapter 22: Policies for Energy System Transformations
Chapter 23: Policies for Energy Access
Chapter 24: Policies for the Energy Technology Innovation System
Chapter 25: Policies for Capacity Building

Next, we propose a structure for the energy system with the help of ontological concepts (Gruber, 1983). In this way, we expand the review of the energy system representation towards classification schemes used for energy and scientific data. We include the Standard International Energy Product Classification by UNSTATS and IRES (SIEC) [2], the Global Change Master Directory Keywords (GCMD 2020) [3], JEL classification [4], and the European Science Vocabulary (EuroSciVoc 2020) [5]. Fig. 1 shows the result for the ontology with the energy system at the center together with its agents (humans, machines), related activities (system monitoring, control, and integration) and available technologies (energy technologies, renewable energy generation technologies, smart technologies, environmental technologies, and others). Table 1 complements the classification and review by linking these classification schemes to a total of 80 energy and other databases which we evaluate in the section below. The databases may belong to one (or several) of concepts of the energy system ontology (Fig. 1,

Tab. 1). Keywords K1-K21 help to further characterize them. See also the details provided database by database in the attached spreadsheet documenting machine assessments.

Fig. 1: Ontological concept of the energy system. See also the repository to this publication for files.

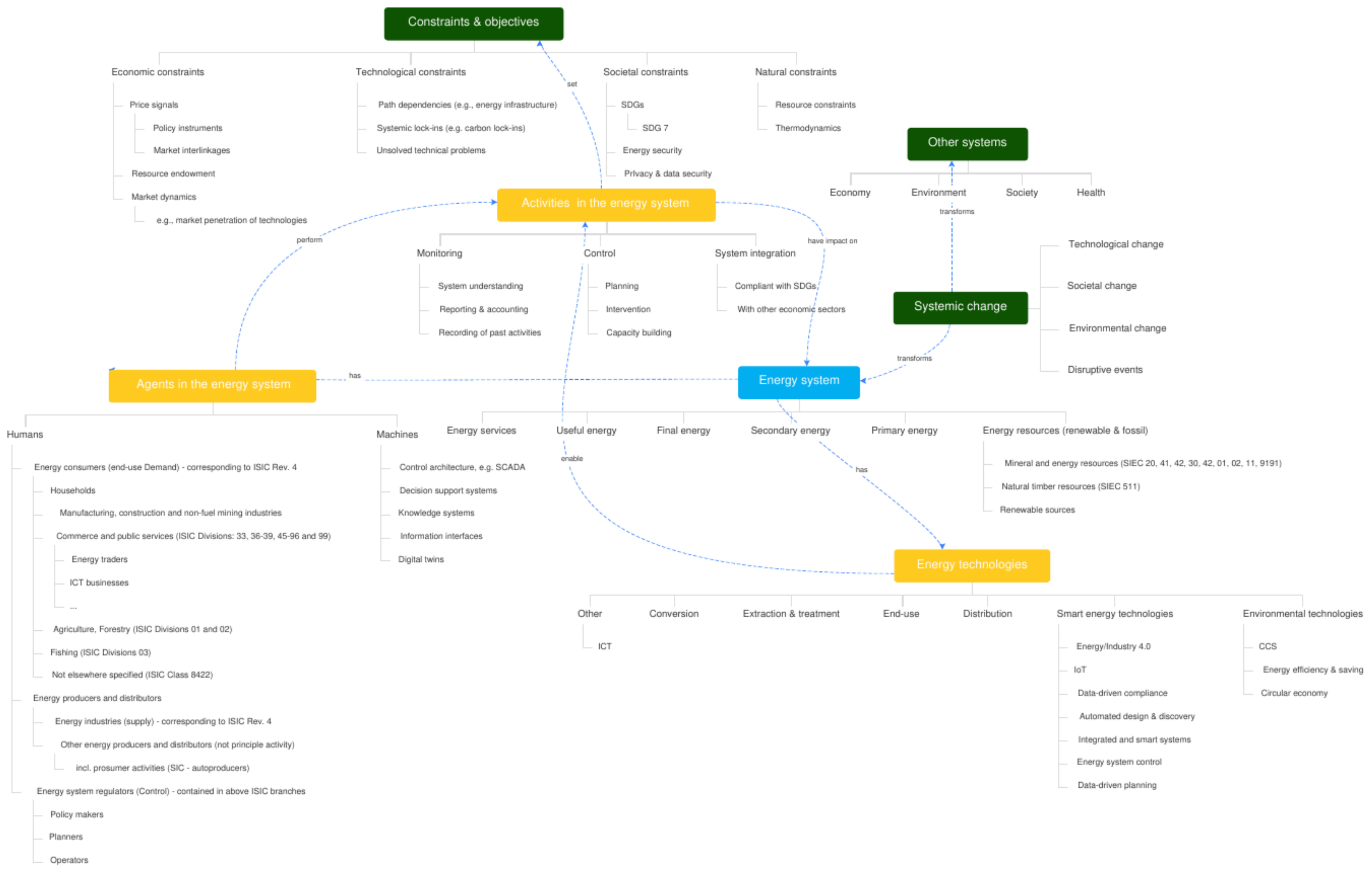
Constraints & objectives
Economic constraints
Price signals
Policy instruments
Market interlinkages
Resource endowment
Market dynamics
e.g., market penetration of technologies
Technological constraints
Path dependencies (e.g., energy infrastructure)
Systemic lock-ins (e.g. carbon lock-ins)
Unsolved technical problems
Societal constraints
SDGs
SDG 7
Energy security
Privacy & data security
Natural constraints
Resource constraints
Thermodynamics
set
Other systems
Economy
Environment
Society
Health
transforms
Activities in the energy system
perform
have impact on
Monitoring
System understanding
Reporting & accounting
Recording of past activities
Control
Planning
Intervention
Capacity building
System integration
Compliant with SDGs
With other economic sectors
Systemic change
Technological change
Societal change
Environmental change
Disruptive events
transforms
Agents in the energy system
has
Energy system
Humans
Energy consumers (end-use Demand) - corresponding to ISIC Rev. 4
Households
Manufacturing, construction and non-fuel mining industries
Commerce and public services (ISIC Divisions: 33, 36-39, 45-96 and 99)
Energy traders
ICT businesses
...
Agriculture, Forestry (ISIC Divisions 01 and 02)
Fishing (ISIC Divisions 03)
Not elsewhere specified (ISIC Class 8422)
Energy producers and distributors
Energy industries (supply) - corresponding to ISIC Rev. 4
Other energy producers and distributors (not principle activity)
incl. prosumer activities (SIC - autoproducers)
Energy system regulators (Control) - contained in above ISIC branches
Policy makers
Planners
Operators
Machines
Control architecture, e.g. SCADA
Decision support systems
Knowledge systems
Information interfaces
Digital twins
Energy services
Useful energy
Final energy
Secondary energy
Primary energy
Energy resources (renewable & fossil)
Mineral and energy resources (SIEC 20, 41, 42, 30, 42, 01, 02, 11, 9191)
Natural timber resources (SIEC 511)
Renewable sources
enable
has
Energy technologies
Other
ICT
Conversion
Extraction & treatment
End-use
Distribution
Smart energy technologies
Energy/Industry 4.0
IoT
Data-driven compliance
Automated design & discovery
Integrated and smart systems
Energy system control
Data-driven planning
Environmental technologies
CCS
Energy efficiency & saving
Circular economy

Table 1: Mapping ontological concepts and 80 databases selected for assessments. Keywords K1-K21 further characterize the concepts. These are K1: demand & supply, K2: system integration, K3: objectives, K4: constraints, K5: data security & privacy, K6: policy process, K7: reporting of transition processes, K8: price signals, K9: resource endowments, K10: generation statistics, K11: energy types, K12: trade, K13: grid status, K14: consumption data, K15: load profiles, K16: control of energy services, K17: system flows incl. storage, K18: signals for plant operation, K19: alerts, K20: modeling, K21: technical parameters & physical constants.

Classification concepts & related keywords	Databases
**Energy system**: Resources; primary, secondary and final energy; energy services.<br><br>*Related keywords: K1, K9, K10, K12, K13, K14, K15, K17.*	OECD Energy Data , IEA Data & Statistics, National energy accounting (Example: National Statistics Poland), CommONEnergy Data Mapper, Satellite Application Facility on Climate Monitoring (CMSAF); Simulations of hourly power output from wind and solar PV farms (Renewables.Ninja); ENTSO-E transparency platform, German Federal Grid Regulator Open Data for Electricity Markets (SMARD); CommONEnergy Data Mapper, Policies to Enforce the Transition to nearly zero energy buildings in EU-27 (Project: Entranze), Global Buildings Performance Network (GBPN), The open source mapping and planning tool for cooling and heating (project: Hotmaps), Mesures d'Utilisation Rationnelle de l'Energie (Mure database), Scenarios of market transition to nearly Zero Energy Buildings (nZEB), EU Building Stock Monitoring (Project: EPISCOPE); EUROSTAT energy balances, IRENA Statistics; European Energy Efficient Building District Database: from data to information to knowledge (Project: ExcEED); Global Energy Institute - US Chamber of Commerce; FAOSTAT - energy use; Joint Research Center (JRC) data catalogue; Heat Roadmap Europe (PETA); Smart Integration of Energy Storages in Local Multi Energy Systems for maximizing the Share of Renewables in Europe's Energy Mix (SmILES); Norwegian Power System Data by STATNET; The WORLD BANK Data Catalog; zenodo Repository; Data Portal of the province Castilla and Leon; EUROSTAT; United Nations Open SDG Data Hub
**Constraints & objectives**: Economic constraints, technological constraints, societal constraints, natural constraints.<br><br>*Related keywords: K3, K4, K5, K6, K8.*	energydata.info (SDG 7), The WORLD BANK Data Catalog, EU Energy Poverty Observatory, Carbon Dioxide Information Analysis Center (CDIAC), United Nations Open SDG Data Hub, European Environment Agency Data, EU Critical Raw Materials - 4th list of CRMs, EUROSTAT electricity prices, NORDPOOL day-ahead electricity prices, NORDPOOL historical market data, International Monetary Fund - Fossil fuel subsidy data; Access to European Union law (EUR-Lex); OECD Database on Policy Instruments for the Environment (PINE); UNEP Material Flow Database; Ireland's Open Data Portal (case: Wicklow Wind Energy Strategy Wicklow County Development Plan 2016 - 2022); https://unstats-undesa.opendata.arcgis.com/; Life Cycle Inventory Database (ecoinvent)
**Agents**: Energy consumers (incl. households, public services and commerce with ICT businesses etc.), energy producers and distributors, energy system regulators<br><br>*Related keywords: K6, K7, K16, K18, K19*	IEA policies database, EMODnet - THE EUROPEAN MARINE OBSERVATION AND DATA NETWORK, Mesures d'Utilisation Rationnelle de l'Energie (Mure database), Access to European Union law (EUR-Lex), European Commission eCERTIS database, Covenant of Mayors for Climate & Energy Europe, OECD Database on Policy Instruments for the Environment (PINE); UK Bus Open Data Service, U.S. National Renewable Energy Laboratory (NREL); ENTSO-E transparency platform; European Environment Agency Data; EUROSTAT electricity prices; FAOSTAT - energy use; International Monetary Fund - Fossil fuel subsidy data; Norwegian Felles datakatalog; Database for support schemes, grid issues and policies about RE sources in EU-28, EFTA etc. (RES LEGAL); Norwegian Power System Data by STATNET; TOMTOM Traffic Index; German Business Registry; Data Portal of the province Castilla and Leon; NORDPOOL day-ahead electricity prices; German Federal Grid Regulator Open Data for Electricity Markets (SMARD)
**Technologies**: Energy t., environmental t., smart technologies & ICT<br><br>*Related keywords: K11*	IIASA Publications Repository (IIASA PURE), Outdoor CPV monitoring (Project: Greco@zenodo), zenodo Repository; Life Cycle Inventory Database (ecoinvent); German Open Government Data Portal (GovData), EU SCIENCE HUB (dataset: PV-GIS); Project Sharewind@zenodo, EMODnet - THE EUROPEAN MARINE OBSERVATION AND DATA NETWORK. EMODnet - THE EUROPEAN MARINE OBSERVATION AND DATA NETWORK**,** Database for biomass and waste (Phyllis); European Energy Efficient Building District Database: from data to information to knowledge (Project: ExcEED); Global Buildings Performance Network (GBPN); The open source mapping and planning tool for cooling and heating (project: Hotmaps); IRENA Statistics; Simulations of

	hourly power output from wind and solar PV farms (Renewables.Ninja); Heat Roadmap Europe (PETA); opendata.swiss Windenergieanlagen; Ireland's Open Data Portal (Wave energy test site);
**Systemic change**: Technological c., societal c., environmental c., disruptive events *Related keywords: K20*	IIASA Data Repository (IIASA DARE); ENERGYDATA.INFO (SDG 7); The International Disaster Database (EM-DAT), Life Cycle Inventory Database (ecoinvent); NORDPOOL historical market data; Statistics on power blackouts (Scientific publication)
**Inter-linkages with other systems** Economy, Environment, Society, Health *Related keywords: K2, K12*	FAOSTAT (land-use), FAOSTAT (energy), FAOSTAT (water), WHO (e.g., air pollution), Satellite Application Facility on Climate Monitoring (CMSAF), EMODnet - THE EUROPEAN MARINE OBSERVATION AND DATA NETWORK, The World Bank Data Catalog, Copernicus Space Component Data Access system (CSCDA); Carbon Dioxide Information Analysis Center (CDIAC); International Monetary Fund (IMF) Data; IIASA Data Repository (IIASA DARE); Nature Scientific Data (Scientific Publication)
Data hubs** not associated with single keywords, **covering large ground in the energy domain	DataCite - Research data repository (>76687 data sets on energy), zenodo Repository (>2226), OECD data (>1028), Joint Research Center (JRC) data catalogue (>1162), U.S. National Renewable Energy Laboratory (NREL) (100), IEA Data & Statistics (unspecified but large number), The WORLD BANK Data Catalog (>1993), Hub - energydata.infoD (558), EUROSTAT (4921); Kaggle repository (>9000); Norwegian Felles datakatalog (>28), Ireland's Open Data Portal (>153); German Open Government Data Portal (GovData) (>4269), United Nations Open SDG Data Hub (tracking of SDG 7 indicators across countries)

2. FAIR/O assessment methods

Different approaches to assess the compliance with the FAIR principles of Wilkinson et al. 2016 [6] exist, but few of them are machine-actionable. Most of them are tools for self-assessment of databases by humans to support improving the FAIRification of data. These tools come in the form of questionnaires and checklists. A comparison of them is done in RDA (2020) [7]. To contrast human-performed and machine-actionable tests, we selected the ARDC DAIR data self-assessment tool (ARDC 2019) [8], comparing it with the FAIR Maturity Evaluation Service (Wilkinson et al. 2019) [9]. Our choice is motivated by the following reasons. In regard to machine-actionable tests, Wilkinson et al. 2019 [9] provides the only comprehensive test at this point. In regard to human-performed tests, we reviewed the different approaches listed in RDA (2020) [7], weighing pro's and con's (Table 2). The reason to select the ARDC FAIR data assessment tool is that it aligns with the FAIR principles, has a good balance between technical and non-technical questions, and enables scoring. In this way, we can directly compare the two assessment perspectives.

To scrutinize our decision, we additionally tested the second-best candidate (SATISFYD) for two databases (Table 3). While both tools indicate ratings for FAIR categories, the questions and answers differ. Hence, the comparison of scores is limited. However, a notable difference in SATISFYD concerns the impact of single factors such as the existence of a license or whether metadata are available without the data itself. This information has an immense impact on the scoring. Furthermore, other factors are underrepresented in comparison to the ARDC tool. Overall, questions are posed in a more general way. At the same time, questions regarding controlled vocabulary, taxonomies and ontologies are very technical and difficult to answer for non-experts. The comparison of these two tools confirms that the ARDC assessment tool aligns better to the FAIR principles, meeting the intention of this study.

Table 2: Brief comparison of FAIR assessment approaches and tools

Method/Tool	Comment
**ARDC:** ANDS-NECTAR-RDS-FAIR data assessment tool	Online assessment tool, developed by the Australian Research Data Commons (ARDC). Designed for manual assessment by the user, **following all FAIR data principles of Wilkinson et al.,2016 [6]**. 12 technical and non-technical questions. Users choose the answers from a drop-down menu. Contains short explanations of FAIR principles and terms. Results from the test are indicated with a progress bar as a new answer is given. Scores are not made explicit. However, one can infer them from the source code. The authors note that the test only serves as an orientation.
**SATIFYD:** the DANS Self-Assessment Tool	Online assessment tool, developed by Data Archiving and Networked Services (DANS) and designed for manual assessment by the user. **Does not follow the FAIR data principles**, only touching upon them. Consists of different questions, e.g., regarding the use of standards to describe data (controlled vocabularies, taxonomies, ontologies). Consists of 12 non-technical questions, thus, easier to understand and answer. Contains short explanations of assessment principles, questions and terms. Indicates rating and scoring by percentages.
**Fair enough?** checklist	Online checklist developed by Data Archiving and Networked Services (DANS). Designed for manual assessment of FAIRness of data(sets) and the trustworthiness of the selected repository. **Does not follow the FAIR data principles**, only touching upon them. Consists of 11 non-technical questions, thus, easier to understand and answer. Contains explanations of terms/questions for corresponding paragraphs. Does not provide rating for FAIR and scoring of overall FAIRness.
**OzNome: The CSIRO 5-star Data Rating** tool	Online assessment tool, developed by Commonwealth Scientific and Industrial Research Organisation, Australia. Designed for manual assessment of FAIRness of data(sets). **Follows the FAIR data principles**. Additionally includes questions on the openness of data (e.g., groups of users with full or limited access to the actual data). Consists of 14 technical and non-technical questions. Contains short explanations of terms/questions. Displays a chart using a 5-star data rating.
**Stewardship Maturity Mix “**Scientific Data Stewardship Maturity Assessment Model Template” (template)	Online self assessment template developed by North Carolina Institute for Climate Studies (CICS-NC), the National Centers for Environmental Information (NCEI), and domain experts. Designed for measuring data stewardship practices and leveraging community best practices and standards. **Does not follow the FAIR data principles.** Key components are: preservability, accessibility, usability, production sustainability, data quality assurance, data quality control & monitoring, data quality assessment, transparency & traceability, and data integrity. Technical and non-technical checks of the components. Contains short explanations of terms/questions. Indicates a self assessment of the maturity level using a 5-level grading scheme from 1 (Ad hoc, not managed) to 5 (optimal/well managed). Does not include scoring relating to FAIR criteria.
**Data Stewardship Wizard** - questionnaire	Online questionnaire to create smart Data Management Plans (DMP) for FAIR Open Science tool, developed by Data Stewardship Wizard (DSW) in cooperation with six research organizations. Designed for DMP development for projects. **Does not follow all FAIR data principles**. Consists of 189 technical and non-technical questions, pertaining to experiment design, data design and planning, capture/ measurement, processing and curation, integration, interpretation, information and insights Does not contain short explanations of terms/questions. Percentage scoring relating to FAIR criteria, “Good DMP Practice” and “Openness”.
Checklist **for Evaluation of Dataset Fitness for Use**	Online checklist developed in cooperation with ICSU World Data System and Research Data Alliance. Designed for manual estimation of FAIRness of data(sets) and data curation. Dataset to be evaluated should be stewarded within a CoreTrustSeal-certified repository. **Does not follow strictly FAIR data principles**, only touching upon them. Consists of 20 non-technical questions, but it is not easy to follow the documentations and checklist. Some criteria are marked with asterisks denoting that assessing will require domain/discipline specific knowledge. Does not contain short explanations of terms/questions. Does not indicate ratings or scoring.
**RDA-SHARC Evaluation,** presentation	Poster presentation created by Research Data Alliance (RDA) and SHAring Rewards & Credits (SHARC). Designed for manual assessment by the user as a decision tree in each FAIR Principle. **Follows the FAIR data principles and Open Science Career Assessment Matrix** designed by EC Working Group on Reward under Open Science. Details on questions in the poster are limited. Unclear if the tool is ready for use. Details are limited. Details are limited.

**WMO-Wide Stewardship Maturity Matrix for Climate Data,** tool	Online assessment tool developed by WMO Stewardship Maturity Matrix for Climate Data (SMM-CD) Working Group. Designed for manual assessment by the user. **Follows "internationally-validated data stewardship best practices"**. Consists of 11 questions. Contains explanations/notes below each question. Indicates rating by maturity level 1 to 5 for the different questions, no overall score.
**Data Use and Services Maturity Matrix,** tool	Online assessment tool developed by MM-Serv Working Group adopting the approach of the NCEI (National Centers for Environmental Information)/CICS-NC Data Stewardship Maturity Matrix (DSMM). Designed for the ESIP-DSC (Data Stewardship Committee)-wide review of the MM-Serv to ensure and improve its quality. **Follows MM-Serv Key Components and Maturity Level Criteria.** Consists of 9 Key Components, each being associated with maturity levels 1 to 5. Contains explanations of terms for corresponding maturity levels. Indicates rating based on maturity levels, no overall score

Table 3: FAIR data tests comparing the tools SATIFYD and ARDC Scorings

Database	DANS	ARDC
Zenodo Repository (Data set 'Greco')	F: 89%, **A: 50%**, I: 100%, R: 87%<br>Total FAIR: 81%<br>O: 100%	F: 94%, **A: 90%,** I: 88%, R: 100%,<br>Total FAIR: 93%<br>O: 100%
EMODnet - THE EUROPEAN MARINE OBSERVATION AND DATA NETWORK	F: 44%, **A: 100%**, I: 75%, R: 74%<br>Total FAIR: 73%<br>O: 67%	F: 53%, **A: 40%**, I: 50%, R: 71%,<br>Total FAIR: 54%<br>O: 67%

2.1 Comparing FAIR/O assessments

We apply and compare two assessment methods. One is the FAIR self-assessment tool developed by the Australian Research Data Commons [8], which is carried out by humans. The other is the FAIR Metrics software developed by Wilkinson et al. 2019 [9]. The table below maps the criteria vis-a-vis with the original assessment criteria by Wilkinson et al. 2016 [6]. For comparing the methods quantitatively, we use the scoring developed by ARDC. We map this score onto the FAIR Metrics questions.

Table 4: Comparison of scores assigned for manual assessments and machine assessments

**FAIR criteria used for manual assessment based on ARDC** with associated points	**Score**	**Maturity indicators used for machine assessment based on FAIR Metrics** - associated scores, assuming equal weights	**Score**
**F1: (Meta)data are assigned a globally unique persistent identifier**			
Does the dataset have any identifiers assigned?<br>A) Globally unique, citable and persistent (e.g. DOI, PURL, ARK or Handle) - 8 points<br>B) Web address (URL) - 3 points<br>C) Local identifier - 1 point<br>D) No identifier - 0 points<br><br>*Note: No separation between data and metadata - likely that users only test the data and not the metadata*	up to 47%	FAIR Metrics Gen2- Unique Identifier (F1): Metric to test if the metadata resource has a unique identifier. This is done by comparing the GUID to the patterns (by regexp) of known GUID schemas such as URLs and DOIs. Known schema are registered in FAIRSharing (https://fairsharing.org/standards/?q=&selected_facets=type_exact:identifier%20schema). 25% of 8/17*100%=2/17*100%<br>FAIR Metrics Gen2 - Identifier Persistence (F1): Metric to test if the unique identifier of the metadata resource is likely to be persistent. Known schema are registered in FAIRSharing (https://fairsharing.org/standards/?q=&selected_facets=type_exact:identifier%20schema). For URLs that don't follow a schema in FAIRSharing we test known URL persistence schemas (purl, oclc, fdlp, purlz, w3id, ark). 25% of 8/17*100%=2/17*100%<br>FAIR Metrics Gen2 - Data Identifier Persistence (F1): Metric to test if the unique identifier of the data resource is likely to be persistent. Known schema are registered in FAIRSharing (https://fairsharing.org/standards/?q=&selected_facets=type_	Scaled to meet up to 8/17 *100%

		exact:identifier%20schema). For URLs that don't follow a schema in FAIRSharing we test known URL persistence schemas (purl, oclc, fdlp, purlz, w3id, ark). 50% of 8/17*100%=4/17*100%	
**F2: Data are described with rich metadata defined by R1.**			
How is the data described with metadata?<br>A) Comprehensively (see suggestion) using a recognized formal machine-readable metadata schema? - 4 p.<br>B) Comprehensively, but in a text-based, non-standard format - 3 points<br>C) Brief title and description - 2 p.<br>D) The data is not described - 0 p.	Not alone, may add 22%	FAIR Metrics Gen2 - Structured Metadata (F2): Tests whether a machine is able to find structured metadata. This could be (for example) RDFa, embedded json, json-ld, or content-negotiated structured metadata such as RDF Turtle 50% of 4/17*100%=2/17*100%<br>FAIR Metrics Gen2 - Grounded Metadata (F2): Tests whether a machine is able to find 'grounded' metadata. i.e. metadata terms that are in a resolvable namespace, where resolution leads to a definition of the meaning of the term. Examples include JSON-LD, embedded schema, or any form of RDF. This test currently excludes XML, even when terms are namespaced. Future versions of this test may be more flexible. 50% of 4/17*100%=2/17*100%	Scaled to meet 4/17 *100%
**F3: Metadata are clearly and explicitly include the identifier of the data described.**			
2) Is the dataset included in all metadata records/files describing the data?<br>A) Yes - 1 point<br>B) No - 0 points<br>*Note: Questions related to F2 and F3 are mixed in order when comparing Wilkinson and ARDC.*<br>*Note: If F1 is answered with 'no' then this question is by default zero.*	May add up to 5%	FAIR Metrics Gen2 - Data Identifier Explicitly In Metadata **(F3):** Metric to test if the metadata contains the unique identifier to the data. This is done by searching for a variety of properties, including foaf:primaryTopic, schema:mainEntity, schema:distribution, sio:is-about, and iao:is-about. schema codeRepository is used for software releases. 50% of 1/17*100%=1/34*100%<br>FAIR Metrics Gen2- Metadata Identifier Explicitly In Metadata (F3): Metric to test if the metadata contains the unique identifier to the metadata itself. This is done using a variety of 'scraping' tools, including DOI metadata resolution, the use of the 'extruct' Python tool, and others...50% of 1/17*100%=1/34*100%	Scaled to meet potential plus of 1/17 * 100%
**F4: (Meta)data are registered and indexed in a search-able resource**			
What type of repository or registry is the metadata record in?<br>A) Data is in one place but discoverable through several registries - 4 points<br>B) Generalist public repository - 2 points<br>C) Domain-specific repository - 2 points<br>D) Local institutional repository - 2 points<br>E) The data is not described in any repository - 0 points	May add up to 23%	FAIR Metrics Gen2 - Searchable in major search engine (F4): Tests whether a machine is able to discover the resource by search, using Microsoft Bing	Up to 4/17 *100%
**A1: Metadata are retrievable by their identifier using a standardized communications protocol.**<br>*A1.1: The protocol is open, free, and universally implementable*<br>*A1.2: The protocol allows for an authentication and authorization procedure where necessary.*			
How accessible is the data?<br>A) Publicly accessible - 5 points<br>B) Fully accessible to persons who meet explicitly stated conditions, e.g., ethics approval for sensitive data - 5 points	A1=(Q1+Q2)/90%	Tested through A1.1 and A1.2.	A1=(Q1+Q2)/90%
	Q1: 51%<br>Q2: Up to 39%	FAIR Metrics Gen2 - Uses open free protocol for data retrieval (A1.1): Data may be retrieved by an open and free protocol. Tests data GUID for its resolution protocol. Currently passes InChI Keys, DOIs, Handles, and URLs.	45% for A1

C) A de-identified/modified subset of the data is publicly accessible - 4 p.<br>D) Embargoed access after a specified date - 3 points<br>E) Unspecified conditional access, e.g., contact the data custodian for access - 2 points<br>F) Access to metadata only - 1 point<br>G) No access to data or metadata - 0 points<br><br>Is the data available online without requiring specialized protocols or tools once access has been approved?<br>A) Standard web service API - 4 points<br>B) Non-standard web service - 3 points<br>C) File download from online location - 2 points<br>D) By individual arrangement - 1 points<br>E) No access to data - 0 points		Recognition of other identifiers will be added upon request by the community. 22.5%<br>FAIR Metrics Gen2 - Uses open free protocol for metadata retrieval (A1.1): Metadata may be retrieved by an open and free protocol. Tests metadata GUID for its resolution protocol. Currently passes InChI Keys, DOIs, Handles, and URLs. Recognition of other identifiers will be added upon request by the community. 22.5%.<br>FAIR Metrics Gen2 - Data authentication and authorization (A1.2): Test a discovered data GUID for the ability to implement authentication and authorization in its resolution protocol. Currently passes InChI Keys, DOIs, Handles, and URLs. It also searches the metadata for the Dublin Core 'accessRights' property, which may point to a document describing the data access process. Recognition of other identifiers will be added upon request by the community. 22.5%<br>FAIR Metrics Gen2 - Metadata authentication and authorization (A1.2): Tests metadata GUID for the ability to implement authentication and authorization in its resolution protocol. Currently passes InChI Keys, DOIs, Handles, and URLs. Recognition of other identifiers will be added upon request by the community. 22.5%	45% for A2
**A2: Metadata are accessible, even when the data are no longer available.**			
Will the metadata record be available even if the data is no longer available?<br>A) Yes - 1 point<br>B) No - 0 points<br>C) Unsure - 0 points	Up to 9%	FAIR Metrics Gen2 - Metadata Persistence (A2): Metric to test if the metadata contains a persistence policy, explicitly identified by a persistencePolicy key (in hashed data) or a http://www.w3.org/2000/10/swap/pim/doc#persistencePolicy predicate in Linked Data. 1/10*100%	10%
**I1: (Meta-)data use a formal, accessible, shared, and broadly applicable language for knowledge representation.**			
What (file) formats is the data available in?<br>A) In a structured, open-standard, machine-readable format - 2 points<br>B) In a structured, open-standard, non-machine-readable format - 1 point<br>C) Mostly in a proprietary format - 0 points	Up to 25%	FAIR Metrics Gen2 - Metadata Knowledge Representation Language (weak) (I1): Maturity Indicator to test if the metadata uses a formal language broadly applicable for knowledge representation. Broad view of what defines a 'knowledge representation language'; in this evaluation, anything that can be represented as structured data will be accepted. 1/16*100%=6.25%<br>FAIR Metrics Gen2 - Metadata Knowledge Representation Language (strong) (I1): Maturity Indicator to test if the metadata uses a formal language broadly applicable for knowledge representation. Broad view of what defines a 'knowledge representation language'; in this evaluation, a knowledge representation language is interpreted as one in which terms are semantically-grounded in ontologies. Any form of RDF will pass this test (including RDF that is automatically extracted by third-party parsers such as Apache Tika). 1/16*100%=6.25%<br>FAIR Metrics Gen2 - Data Knowledge Representation Language (weak) (I1): Maturity Indicator to test if the data uses a formal language broadly applicable for knowledge representation. Test takes a broad view of what defines a 'knowledge representation language'; in this evaluation, a knowledge representation language is interpreted as one in which terms are semantically-grounded in ontologies. Any form of structured data will pass this test. 1/16*100%=6.25%	25%

		FAIR Metrics Gen2 - Data Knowledge Representation Language (strong) (I1): Maturity Indicator to test if the data uses a formal language broadly applicable for knowledge representation. This particular test takes a broad view of what defines a 'knowledge representation language'; in this evaluation, a knowledge representation language is interpreted as one in which terms are semantically-grounded in ontologies. Any form of ontologically-grounded linked data will pass this test. 1/16*100%=6.25%	
**I2: (Meta)data use vocabularies that follow FAIR principles.**			
What best describes the types of vocabularies/ontologies/tagging schemas used to define the data elements?<br>A) Standardised, open and universal using resolvable global identifiers linking to explanations - 3 points<br>B) Standardised vocabularies/ ontologies/ schemas without global identifiers - 2 points<br>C) No standards have been applied in the description of data elements - 1 point<br>D) Data elements are not described - 0 points<br>*Note: FAIR is not tested here!*	Up to 37%	FAIR Metrics Gen2 - Metadata uses FAIR vocabularies (weak) (I2): Maturity Indicator to test if the linked data metadata uses terms that resolve. This tests only if they resolve, not if they resolve to FAIR data, therefore is a somewhat weak test. 1/8*100%=12.5%<br>FAIR Metrics Gen2 - Metadata uses FAIR vocabularies (strong) (I2): Maturity Indicator to test if the linked data metadata uses terms that resolve to linked (FAIR) data. Additionally 2/8*100%=25%.	Either 0 or 12.5% or 37.5%
**I3: (Meta)data include qualified references to other metadata.**			
How is the metadata linked to other data and metadata (to enhance context and clearly indicate relationships)?<br>A) Metadata is represented in a machine-readable format, e.g., in a linked data format such as resource-descriptive framework (RDF) - 3 points<br>B) The metadata record includes URI links to related metadata, data, and definitions - 2 points<br>C) There are no links to other metadata - 0 points	Adds up to 38%	FAIR Metrics Gen2 - Metadata contains qualified outward references) (I3): Maturity Indicator to test if the metadata links outward to third-party resources. It only tests metadata that can be represented as Linked Data. 3/8*100%=37.5%	Either 0% or 37.5%
**R1: (Meta)data are richly described with a plurality of accurate and relevant attributes.**<br>*R1.1: (Meta)data are released with a clear and accessible data usage license.*<br>*R1.2: (Meta)data are associated with detailed provenance.*<br>*R1.3: (Meta)data meet domain-relevant community standards.*			
Which of the following best describes the license/usage rights attached to the data?<br>A) Standard machine-readable license, e.g., creative commons - 4 points<br>B) Standard text-based license - 3 points<br>C) Non-standard machine-readable license (clearly indicating under what conditions the data may be reused) - 3 points	Up to 57%	FAIR Metrics Gen2 - Metadata Includes License (strong) (R1.1): Maturity Indicator to test if the linked data metadata contains an explicit pointer to the license. Tests: xhtml, dvia, dcterms, cc, data.gov.au, and Schema license predicates in linked data, and validates the value of those properties. 4/7*100%=57%<br>FAIR Metrics Gen2 - Metadata Includes License (weak) (R1.1): Maturity Indicator to test if the metadata contains an explicit pointer to the license. This 'weak' test will use a case-insensitive regular expression, and scan both key/value style metadata, as well as linked data metadata. Tests: xhtml, dvia, dcterms, cc, data.gov.au, and Schema	R1.1 + R1.2. Either 0% or 43% or 57%

D) Non-standard text-based license - 2 points<br>E) No license - 0 points		license predicates in linked data, and validates the value of those properties. 3/7*100%=43%<br><br>*Note: Here it is tested whether the license is correct AND machine-readable as in A). Thus, it is much more than what is tested by ARDC (even in the weak case).*	
How much provenance information has been captured to facilitate data re-use?<br>A) Fully recorded in a machine-readable format - 3 p.<br>B) Fully recorded in a text format - 2 points<br>C) Partially recorded - 1 points<br>D) No provenance information is recorded - 0 points	Up to 43%	-	N/A
**O1: Openness - How open is the data?**			
A) Publicly accessible by humans and machines alike by standard protocol (open, no log in etc. required)<br>B) Publicly accessible but requires human intervention (e.g., may require login to download etc.)<br>C) Fully accessible to persons who meet explicitly stated conditions, (e.g., ethics approval for sensitive data)<br>D) Data are not open	A) 100%<br>B) 2/3*100%<br>C) 1/3*100%<br>D) 0%		

2.2 FAIR assessment tool by ARDC

The score of the ARDC assessment tool is based on points associated with the answers given. Twelve questions Q1-Q12 are posted. Answers are provided as options from a drop-down menu (between 2-7 options). Options are connected to points, and different answers may be similarly weighted. However, the highest points are connected with the first choice of an answer offered per question. The maximum scores that can be achieved for each FAIR criteria are F=17, A=10, I=8, R=7. The total score is calculated using the following formula:

- Formula: sum_i sum_j w_{ij} q_{ij} / sum_i \max w_{ij} with $i \element{F,A,I,R}$, and w being the weights above and q being the answers to i=number of question and j=number of answer options. q takes either 0 (not being the answer) or 1 (being the answer).
- The following weights apply. They are taken from the points associated with answers:

Q1-F1: 0,1,3,**8**<br>Q2-F2: 0,2,3,**4**<br>Q3-F3: 0,**1**	Q4-F4: 0,2,2,2,**4**<br>Q5-A1: 0,1,2,3,4,5,**5**<br>Q6-A1: 0,1,2,3,**4**	Q7-A2: 0,0,**1**<br>Q8-I1: 0,1,**2**<br>Q9-I2: 0,1,2,**3**	Q10-I3: 0,2,**3**<br>Q11-R1: 0,2,3,3,**4**<br>Q12-R1: 0,1,2,**3**

- Note an exception in Q1. If there is no identifier in the dataset, Q3 must also default to zero.
- The score is calculated from % scores of each FAIR component. All are weighted equally (25%).

2.3 Machine-based assessment of FAIR criteria

The machine-based assessments were developed by Wilkinson et al. (2019) [9]. They follow the FAIR principles in detail. Once installed, the software requires an input of an GUI ID to be tested along with the identifier of the person testing (e.g., ORCID). The tests take a few minutes and return two types of

results, one being a summary picture (see Fig. 2) and the other being the returns from the software processing the GUI ID in the form of a '0' (failure of test) or '1' (pass of test). A key for the software to return positive results is the existence of sufficient metadata details.

Fig. 2 Screenshot from summary result of testing the instance from zenodo.

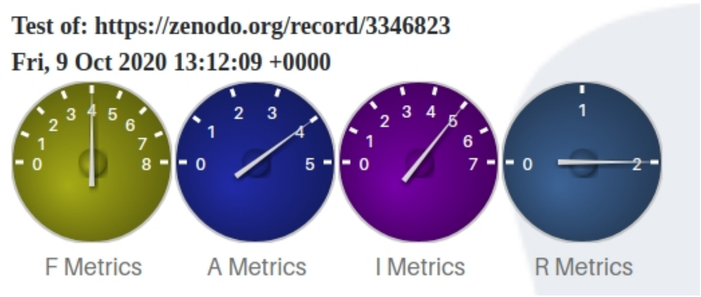

For comparing machine-based assessments of databases with manual assessments, we have to map the questions onto each other, using the same scoring and weighting of answers in each of the FAIR categories. The table below shows the assumptions in line with scores given in the ARDC-based manual assessment algorithm (Section 2.2) and their mapping onto the machine assessment (Table 5).

Table 5: Weights used to map results from machine assessments with manual assessments. The weights are used to calculate the scores in each of them.

**Test #**	**Weight**	**Answer** (example)	**Test #**	**Weight**	**Answer** (example)	**Test #**	**Weight**	**Answer** (example)	**Test #**	**Weight**	**Answer** (example)
1	0.12	0	1	0.225	0	1	0.0625	0	1	4/7	0
2	0.12	0	2	0.225	0	2	0.0625	0	2	3/7	0
3	0.24	1	3	0.225	1	3	0.0625	1			
4	0.12	1	4	0.225	0	4	0.0625	1			
5	0.12	1	5	0.1	1	5	0.125	1			
6	0.03	1				6	0.25	1			
7	0.03	0				7	0.375	1			
8	0.24	1									
**F score:**	0.74		**A score:**	0.33		**I score:**	0.88		**R score:**	0	
**Total:**						**0.48**					

3. Results

3.1 Aggregated summary for manual vs. machine assessments

**Name of database**	**Manual assessment**	**Machine assessment**
Satellite Application Facility on Climate Monitoring (CMSAF)	F: 82%, A: 80%, I: 63%, R: 29%<br>Total FAIR: 64%; O: 67%	F: 74%, A: 33%, I: 88%, R: 0%<br>Total FAIR: 48%
CommONEnergy Data Mapper	F: 41%, A: 70%, I: 38%, R: 43%<br>Total FAIR: 48%; O: 67%	F: 50%, A: 33%, I: 13%, R: 0%<br>Total FAIR: 24%

Copernicus Space Component Data Access system (CSCDA)	F: 65%, A: 90%, I: 88%, R: 86% Total FAIR: 82%; O: 100%	*F: 0%, A: 0%, I: 0%, R: 0% Total FAIR: 0%*
Covenant of Mayors	F:47%, A: 70%, I: 50%, R: 29% Total FAIR: 49%; O: 67%	F: 50%, A: 33%, I: 88%, R: 0% Total FAIR: 43%
DataCite	F: 59% , A: 90%, I: 75% , R: 57%, Total FAIR: 70%; O: 33%	F: 50%, A: 33%, I: 13%, R: 0% Total FAIR: 24%
EMODnet - THE EUROPEAN MARINE OBSERVATION AND DATA NETWORK	F: 53%, A: 40%, I: 50%, R: 71%, Total FAIR: 54%; O: 67%	F: 24%, A:33%, I: 0%, R: 0%, Total FAIR: 14%
Policies to Enforce the Transition to nearly zero energy buildings in EU-27 (Project: Entranze)	F: 47%, A: 70%, I: 38%, R: 43% Total FAIR: 49.5%; O: 67%	F: 24%, A:33%, I: 0%, R: 0%, Total FAIR: 14%
ENTSO-E transparency platform	F: 41%, A: 70%, I: 0%, R: 0% Total FAIR: 28%; O: 67%	F: 50%, A:33%, I: 25%, R: 0%, Total FAIR: 27%
EUR-Lex (Document: Directive 95/46/EC)	F: 47%, A: 80%, I: 63%, R: 57% total FAIR: 62%; O: 67%	F: 50%, A:33%, I: 25%, R: 0%, Total FAIR: 27%
Global Buildings Performance Network (GBPN)	F: 70%, A: 70%, I: 50%, R: 14% Total FAIR: 51%; O: 67%	F: 50%, A:33%, I: 25%, R: 0%, Total FAIR: 27%
The open source mapping and planning tool for cooling and heating (project: Hotmaps)	F: 47%, A: 70%, I: 50%, R: 86% Total FAIR: 63%; O: 67%	F: 24%, A:33%, I: 0%, R: 0%, Total FAIR: 14%
IEA Policy database	F: 47%, A: 50%, I: 38%, R: 57% Total FAIR: 48%; O: 100%	F:50%, A: 33%, I: 13%, R:0%, Total FAIR: 24 %
IEA World Energy Statistics and Balances	F: 47%, A:50%, I: 38%, R: 57%, Total FAIR: 48%; O: 0%	F:74%, A: 33%, I: 88%, R:0%, Total FAIR: 48 %
JRC data catalogue	F: 53%, A: 70%, I: 63%, R: 57% Total FAIR: 61%; O: 100%	F:62%, A: 78%, I: 88%, R:0%, Total FAIR: 57 %
JRC data catalogue - data collection	F: 47%, A:100%, I: 38%,R: 43%, Total FAIR: 57%, O: 100%	F:50%, A: 33%, I: 13%, R:0%, Total FAIR: 24 %
JRC data catalogue - data set	F: 47%, A:80%, I: 25%, R: 43%, Total FAIR: 49%, O:67%	F:62%, A: 78%, I: 88%, R:0%, Total FAIR: 57%
Mesures d'Utilisation Rationnelle de l'Energie (Mure database)	F: 47%, A: 70%, I: 63%, R: 29% Total FAIR: 52%, O: 67%	F:50%, A: 33%, I: 13%, R:0%, Total FAIR: 24%
Kaggle Repository (data set)	F:41%, A: 80%, I: 25%, R: 43% Total FAIR: 47%; O: 67%	F: 74%, A: 78%, I: 88%, R: 0% Total FAIR: 60%
Simulations of hourly power output from wind and solar PV farms (Renewables.Ninja)	F: 76%, A: 70%, I: 75%, R: 71% Total FAIR: 73%; O: 67%	F: 24%, A: 33%, I: 0%, R: 0% Total FAIR: 14%
U.S. National Renewable Energy Laboratory (NREL) data catalog	F: 100%, A: 90%, I: 50%,R: 29% Total FAIR: 67%; O: 100%	F: 62%, A: 33%, I: 88%, R: 0% Total FAIR: 45%
Scenarios of market transition to nearly Zero Energy Buildings (nZEB)	F: 53%, A: 60%, I: 38%, R: 0% Total FAIR: 38%; O: 67%	F: 24%, A: 33%, I: 0%, R: 0% Total FAIR: 14%
OECD hub (energy data)	F: 70%, A: 90%, I: 75%,R: 100% Total FAIR: 84%; O: 100%	F: 50%, A: 33%, I: 13%, R:0%, Total FAIR: 24%
OECD hub (data set)	F: 77% , A: 70%, I: 38% ,R: 29% Total FAIR: 53%; O: 67%	F: 50% , A: 33%, I: 13% , R: 0%; Total FAIR: 24%
Heat Roadmap Europe (PETA)	F: 59%, A: 60%, I: 38%, R: 0% Total FAIR: 39%; O: 67%	F: 50%, A: 33%, I: 50%, R:0%, Total FAIR: 33%
Database for support schemes, grid issues and policies about RE sources in EU-28, EFTA etc. (RES LEGAL)	F: 47%, A: 80%, I: 38%, R: 29% Total FAIR: 49%; O: 100%	F: 24% , A: 33%, I: 0% , R: 0% Total FAIR: 14%
EU Building Stock Monitoring (Project: EPISCOPE)	F: 47%, A: 70%, I: 25%, R: 43% Total FAIR: 46%; O: 67%	F: 24% , A: 33%, I: 0% , R: 0% Total FAIR: 14%
Zenodo Repository (Data set 'Greco')	F: 94%, A: 90%, I:88%,R: 100%, Total FAIR: 93%; O: 100%	F:62%, A: 78%, I: 88%, R:100%, Total FAIR: 82%
Zenodo Repository (Data set 'Sharewind')	F: 100%, A: 80%, I: 63%, R:57% Total FAIR: 75%; O: 100%	F: 38%,A: 33%,I: 88%, R: 100%; Total FAIR: 65%

3.2 Results from individual manual assessments

The figures below contrast the assessment results before and after a joint briefing on how to interpret the FAIR assessment criteria. On the left hand side, black and red entries are shown, where black is the result for a single manual assessment and red shows the results for at least two assessments of the same database. If they are not connected by a line, assessments are on top of each other (agreement). The right hand side compares the results from manual assessments with those obtained through the machine-based assessment. Note that the results for interoperability are reported in the main document.

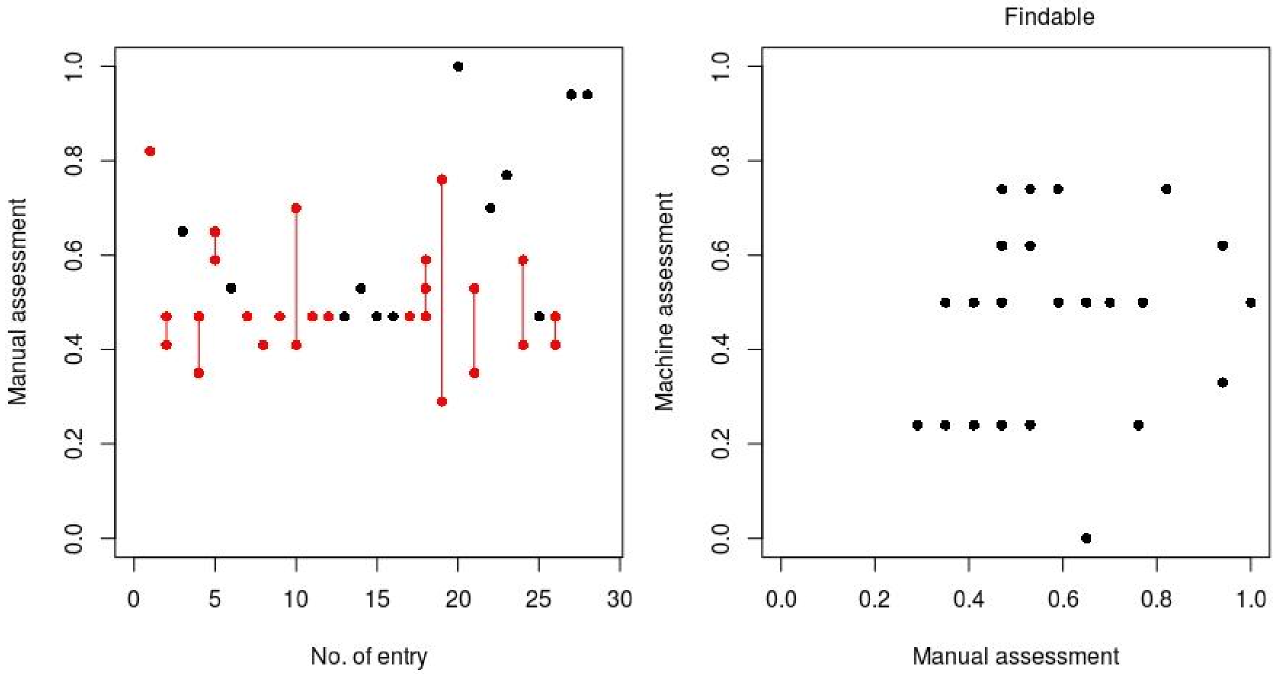

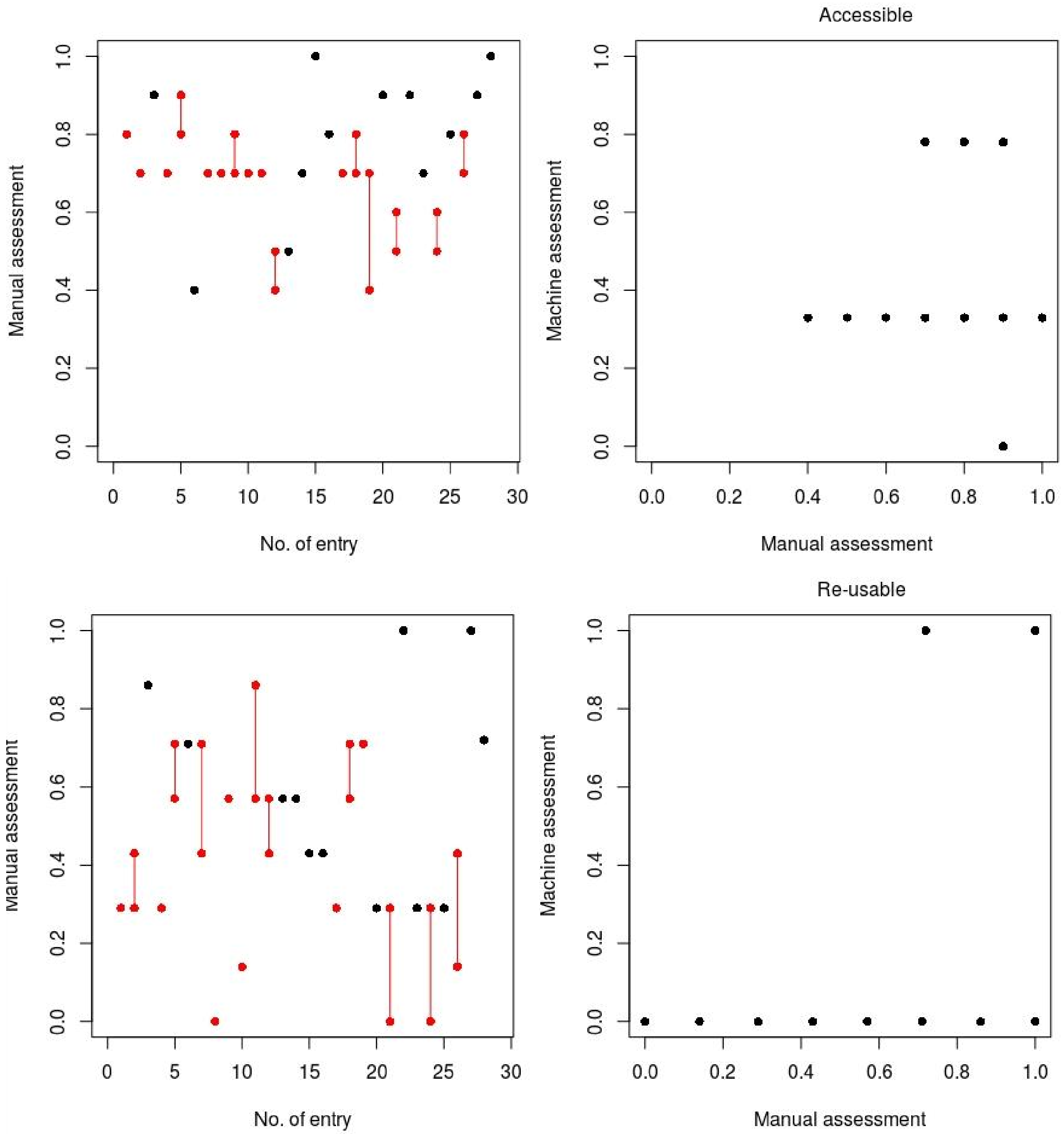

Accessible
Re-usable
Manual assessment
Machine assessment
No. of entry
0.0
0.2
0.4
0.6
0.8
1.0
0
5
10
15
20
25
30

Database	Assessor	Q1	Q2	Q3	Q4	Q5	Q6	Q7	Q8	Q9	Q10	Q11	Q12	F	A	I	R	Total	Comments	Differences to assessment before briefing	Q1	Q2	Q3	Q4	Q5	Q6	Q7	Q8	Q9	Q10	Q11	Q12	F	A	I	R	Total
nZEB	CBS	3	3	0	0	5	0	0	0	2	2	0	0	35	50	50	29	41			5	2	0	2	5	1	0	1	2	0	0	0	53	60	38	0	38
OECD data	CBS	3	2	0	2	5	4	0	2	1	2	0	1	41	90	63	14	52			3	4	1	4	5	4	0	2	2	2	4	3	70	90	75	100	84
OECD energy data	CBS	5	2	0	2	5	4	0	2	1	2	0	2	53	90	63	29	59			8	3	0	2	5	2	0	2	1	0	2	0	77	70	38	29	53
PETA	CBS	3	2	0	2	5	0	0	0	1	0	0	2	41	50	13	29	33			5	3	0	2	5	1	0	1	2	0	0	0	59	60	38	0	39
MURE	MB	3	4	1	2	1	3	1	1	2	0	2	0	59	50	38	29	44			3	3	0	2	5	2	0	1	2	2	0	2	47	70	63	29	52
Nat. energy stats.	MB	3	2	1	2	5	2	1	1	0	2	0	1	47	80	38	14	45			-	-	-	-	-	-	-	-	-	-	-	-	-	-	-	-	-
Kaggle – repository	MB	3	2	1	2	2	2	1	1	1	0	2	1	47	50	25	43	41			-	-	-	-	-	-	-	-	-	-	-	-	47	80	75	71	68
Kaggle – repository	MEB	3	3	1	2	5	2	0	2	2	2	3	2	53	70	75	71	67			-	-	-	-	-	-	-	-	-	-	-	-	-	-	-	-	-
Kaggle – data set	MB	3	4	1	2	5	3	0	1	2	2	4	1	59	80	63	71	68			-	-	-	-	-	-	-	-	-	-	-	-	-	-	-	-	-
Kaggle - data set	MEB	5	2	0	2	5	2	-	2	2	2	2	2	53	70	75	57	64			-	-	-	-	-	-	-	-	-	-	-	-	-	-	-	-	-
Ninja	MB	3	2	0	0	2	2	0	1	1	0	4	1	29	40	25	71	41			8	3	0	2	5	2	0	2	2	2	4	1	76	70	75	71	73
Ninja	MEB	8	2	1	2	5	2	0	2	2	2	3	2	76	70	75	71	73			-	-	-	-	-	-	-	-	-	-	-	-	-	-	-	-	-
SMILES	MC	1	0	0	2	5	2	1	0	1	0	0	0								3	4	0	2	5	2	0	2	2	0	0	1	53	70	50	14	47
Tabula	MC	1	3	1	2	5	2	1	0	1	0	0	1								3	3	0	2	5	2	0	0	2	-	2	1	47	70	25	43	46
Zenodo	MC	8	4	1	4	5	4	1	2	3	3	4	3								-	-	-	-	-	-	-	-	-	-	-	-	-	-	-	-	-
Zenodo – Dataset GRECO	MC	8	4	1	4	5	4	1	2	2	3	4	3								8	4	0	4	5	4	0	2	2	3	4	3	94	90	88	100	93
Zenodo – Dataset SHAREWIND	MC	8	4	1	4	5	4	1	2	2	3	4	3								-	-	-	-	-	-	-	-	-	-	-	-	-	-	-	-	-
CMSAF	MP	8	3	1	2	5	2	1	1	2	2	0	2	82	80	63	29	64			8	3	1	2	5	2	1	1	2	2	0	2	82	80	63	29	64

COMONEnergy	MP	3	3	0	2	5	2	0	1	2	2	0	2	47	70	63	29	52			3	2	0	2	5	2	0	1	2	0	2	1	41	70	38	43	48
Copernicus – CAMS Climate Forcing	MP	3	4	1	4	5	3	0	2	3	3	3	3	71	80	100	86	84			3	4	0	4	5	4	0	2	2	3	4	2	65	90	88	86	83
Covenant of Mayors	MP	3	0	1	2	5	2	0	1	2	2	0	2	35	70	63	29	49			3	3	0	2	5	2	0	0	2	2	0	2	47	70	50	29	49
Datacite – Repository	MP	3	3	1	4	5	3	0	2	3	3	3	2	65	80	100	71	79			3	4	1	2	5	4	0	2	2	2	3	1	59	90	75	57	70
MURE	MEB	3	3	0	2	5	2	0	2	2	0	2	0	47	70	50	29	49			3	3	0	2	5	2	0	1	2	2	0	2	47	70	63	29	52
National Energy Statistics	MEB	3	3	0	2	5	2	0	1	2	2	0	1	47	70	63	14	49			-	-	-	-	-	-	-	-	-	-	-	-	-	-	-	-	-
IUR LEX	MHD	3	3	0	2	5	2	0	1	2	2	4	0	47	70	63	57	59			3	3	0	2	5	3	0	1	2	2	2	2	48	80	63	57	62
ENTSO-E	MHD	3	2	0	2	5	2	0	0	0	0	0	0	41	70	0	0	28			3	2	0	2	5	2	0	0	0	0	0	0	41	70	0	0	28
ENTRANZE	MHD	3	3	0	2	5	2	0	1	2	0	2	1	47	70	38	71	50			3	3	0	2	5	2	0	1	2	-	2	1	47	70	38	43	50
EMODnet - dataset	MHD	3	4	1	2	5	2	0	2	2	2	3	2	59	70	75	71	69			-	-	-	-	-	-	-	-	-	-	-	-	-	-	-	-	-
EMODnet - hub	MHD	3	3	1	2									53							3	4	0	2	2	1	1	2	2	0	4	1	53	40	50	71	54
EXCEED	MK	1	2	0	0	2	1	0	1	2	0	0	0	18	30	38	0	22			1	3	0	0	2	1	0	1	2	-	0	0	23	30	38	0	23
GBPN	MK	3	2	0	2	5	2	0	1	1	2	0	1	41	70	50	14	44			8	2	0	2	5	2	0	1	1	2	0	1	70	70	50	14	51
Global Energy Institute	MK	3	3	1	2	4	2	1	1	1	2	0	1	53	70	50	14	50			-	-	-	-	-	-	-	-	-	-	-	-	-	-	-	-	-
HOTMAPS	MK	3	3	0	2	5	2	0	2	2	0	3	1	47	70	50	57	63			3	3	0	2	5	2	0	2	2	0	4	2	47	70	50	86	63
IEA - Policy database	MK	3	3	0	2	2	2	0	1	1	0	2	1	47	40	25	43	39			3	3	0	2	2	2	1	1	2	0	3	1	45	50	38	57	48

The following tables give the results from manual assessments, including comments to motivate choices.

Name of database	Answers to Q1-Q12 (ARDC)	Score	Criteria
Satellite Application Facility on Climate	Q1: Globally unique, citable and persistent (e.g. DOI, PURL, ARK or Handle)	8	
	Q2: Comprehensively, but in a text-based, non-standard format	3	

Monitoring (CMSAF)	Q3: Yes	1	
	Q4: Domain-specific repository	2	
		14/17=**82%**	**F score**
	Q5: Fully accessible to person who meet explicitly stated conditions, e.g. ethics approval for sensitive data	5	
	Q6: File download from online location	2	
	Q7: Yes	1	
		8/10=**80%**	**A score**
	Q8: In a structured, open standard, non-machine-readable format	1	
	Q9: Standardised vocabularies/ontologies/schema without global identifiers	2	
	Q10: The metadata record includes URI links to related metadata, data and definitions	2	
		5/8=**63%**	**I score**
	Q11: No license	0	
	Q12: Fully recorded in a text format	2	
		2/7=**29%**	**R score**
		82% x 0.25 + 80% x 0.25 + 63% x 0.25 + 29% x 0.25 = **64%**	**FAIR total score**
	O1: Publicly accessible but requires human intervention (e.g., may require login to download etc.)	**67%**	**Openness score**

**Name of database**	**Answers to Q1-Q12 (ARDC)**	**Score**	**Criteria**
CommONErgy data Mapper	Q1: Web address (URL)	3	
	Q2: Brief title and description	2	
	Q3: No	0	
	Q4: Generalist public repository	2	
		7/17=**41%**	**F score**
	Q5: Publicly accessible	5	
	Q6: File download from online location	2	
	Q7: Unsure	0	
		7/10=**70%**	**A score**
	Q8: In a structured, open standard, non-machine-readable format	1	
	Q9: Standardised vocabularies/ontologies/schema without global identifiers -	2	
	Q10: There are no links to other metadata		
		0	
		3/8=**38%**	**I score**
	Q11: Non-standard text-based license	2	
	Q12: Partially recorded	1	
		3/7=**43%**	**R score**
		41% x 0.25 + 70% x 0.25 + 38% x 0.25 + 43% x 0.25 = **48%**	**FAIR total score**
	B) Publicly accessible but requires human intervention (e.g., may require login to download etc.)	2/3=**67%**	**Openness Score**

Name of database	Answers to Q1-Q12 (ARDC)	Score	Criteria
Copernicus Space Component Data Access System (CSCDA)	Q1: Web address (URL)	3	
	Q2: Comprehensively (see suggestion) using a recognized, formal machine-readable metadata schema	4	
	Q3: No	0	
	Q4: Data is in one place but discoverable through several registries	4	
		11/17=**65%**	**F score**
	Q5: Fully accessible to person who meet explicitly stated conditions, e.g. ethics approval for sensitive data	5	
	Q6: Standard web service API (e.g. OGC	4	
	Q7: Unsure	0	
		9/10=90**%**	**A score**
	Q8: In a structured, open standard, machine-readable format	2	
	Q9: Standardized vocabularies/ontologies/schema without global identifiers	2	
	Q10: Metadata is represented in a machine readable format, e.g. in a linked data format such as Resource Description Framework (RDF)	3	
		7/8=88**%**	**I score**
	Q11: Standard machine-readable license (e.g. Creative Commons)	4	
	Q12: Fully recorded in a text format	2	
		6/7=86**%**	**R score**
		65% x 0.25 + 90% x 0.25 + 88% x 0.25 + 86% x 0.25 = **83%**	**FAIR total score**
	O1: Publicly accessible by humans and machines alike by standard protocol (open, no log in etc. required)	**100%**	**Openness score**

Name of database	Answers to Q1-Q12 (ARDC)	Score	Criteria
Covenant of Mayors	Q1: Web address (URL)	3	
	Q2: Comprehensively, but in a text-based, non-standard format	3	
	Q3: No	0	
	Q4: Local institutional repository	2	
		8/17=**47%**	**F score**
	Q5: Publicly accessible	5	
	Q6: File download from online location	2	Open for discussion, data not explicitly downloadable, but copyable
	Q7: Unsure	0	
		7/10=**70%**	**A score**
	Q8: Mostly in a proprietary format	0	

	Q9: Standardized vocabularies/ontologies/schema without global identifiers	2	
	Q10: The metadata record includes URI links to related metadata, data and definitions	2	
		4/8=**50%**	**I score**
	Q11: No license	0	
	Q12: Fully recorded in a text format	2	
		2/7=**29%**	**R score**
		47% x 0.25 + 70% x 0.25 + 50% x 0.25 + 29% x 0.25 = **49%**	**FAIR total score**
	O1: Publicly accessible but requires human intervention (e.g., may require login to download etc.)	**67%**	**Openness score**

**Name of database**	**Answers to Q1-Q12 (ARDC)**	**Score**	**Criteria**
DataCite	Q1: Web address (URL)	3	
	Q2: Comprehensively (see suggestion) using a recognized formal machine-readable metadata schema	4	
	Q3: Yes	1	
	Q4: Generalist public repository	2	
		10/17=**59%**	**F score**
	Q5:Fully accessible to persons who meet explicitly stated conditions, e.g., ethics approval for sensitive data	5	
	Q6: Standard web service API (e.g. OGC)	4	
	Q7: Unsure	0	
		9/10**=90%**	**A score**
	Q8: In a structured, open-standard, machine-readable format	2	
	Q9:Standardized vocabularies/ontologies/schema without global identifiers	2	
	Q10: The metadata record includes URI links to related metadata, data and definitions	2	
		6/8**=75%**	**I score**
	Q11: Standard text-based license	3	
	Q12: Partially recorded	1	
		4/7=**57%**	**R score**
		59% x 0.25 + 90% x 0.25 + 75% x 0.25 + 57% x 0.25 = **70%**	**FAIR total score**
	O1: Fully accessible to persons who meet explicitly stated conditions, (e.g., ethics approval for sensitive data, membership fees)	**33%**	**Openness score**

Name of database	Answers to Q1-Q12 (ARDC)	Score	Criteria
EMODnet - THE EUROPEAN MARINE OBSERVATION AND DATA NETWORK	Q1: Web address (URL)	3	
	Q2: Comprehensively (see suggestion) using a recognised, formal machine-readable metadata schema	4	
	Q3: No	0	
	Q4: Local institutional repository	2	
		9/17=**53%**	**F score**
	Q5: Unspecified conditional access e.g. contact the data custodian for access	2	
	Q6: By individual arrangement	1	
	Q7: Yes (the metadata is present but for the data itself contact custodian)	1	
		4/10=**40%**	**A score**
	Q8: In a structured, open standard, non-machine-readable format	2	
	Q9: Standardized vocabularies/ontologies/schema without global identifiers	2	
	Q10: There are no links to other metadata	0	
		4/8=**50%**	**I score**
	Q11: Standard machine-readable license (e.g. Creative Commons)	4	
	Q12: Partially recorded	1	
		5/7=**71%**	**R score**
		53% x 0.25 + 40% x 0.25 + 50% x 0.25 + 71% x 0.25 = **54%**	**FAIR total score**
	O1: Publicly accessible but requires human intervention (e.g., may require login to download etc.)	**67%**	**Openness score**

Name of database	Answers to Q1-Q12 (ARDC)	Score	Criteria
Policies to Enforce the Transition to Nearly Zero Energy Buildings in EU-27 (Project: ENTRANZE)	Q1: Web address (URL)	3	
	Q2: Comprehensively, but in a text-based, non-standard format	3	
	Q3: No	0	
	Q4: Generalist public repository	2	
		8/17=**47%**	**F score**
	Q5: Publicly accessible	5	
	Q6: File download from online location	2	
	Q7: Unsure	0	
		7/10=**70%**	**A score**
	Q8: In a structured, open standard, non-machine-readable format	1	
	Q9: Standardized vocabularies/ontologies/schema without global identifiers -	2	
	Q10: There are no links to other metadata		
		3/8=**38%**	**I score**
	Q11: Non-standard text-based license	2	

	Q12: Partially recorded	1	
		3/7=**43%**	**R score**
		47% x 0.25 + 70% x 0.25 + 38% x 0.25 + 43% x 0.25 = **49.5%**	**FAIR total score**
	O1: Publicly accessible but requires human intervention (e.g., may require login to download etc.)	2/3=67%	**Openness Score**

**Name of database**	**Answers to Q1-Q12 (ARDC)**	**Score**	**Criteria**
ENTSO-E Transparency Platform	Q1: Web address (URL)	3	
	Q2: Brief title and description	2	
	Q3: No	0	
	Q4: Local institutional repository	2	
		7/17=**41%**	**F score**
	Q5: Publicly accessible	5	
	Q6: File download from online location	2	
	Q7: No	0	
		7/10=**70%**	**A score**
	Q8: Mostly in a proprietary format	0	
	Q9: Data elements not described	0	
	Q10: There are no links to other metadata	0	
		0/8=**0%**	**I score**
	Q11: No license	0	
	Q12: No provenance information is recorded	0	
		0/7=**0%**	**R score**
		41% x 0.25 + 70% x 0.25 + 0% x 0.25 + 0% x 0.25 = **28%**	**FAIR total score**
	O1: Publicly accessible but requires human intervention (e.g., may require login to download etc.)	**67%**	**Openness score**

**Name of database**	**Answers to Q1-Q12 (ARDC)**	**Score**	**Criteria**
EUR-Lex (Document: Directive 95/46/EC)	Q1: Web address (URL)	3	
	Q2: Comprehensively, but in a text-based, non-standard format	3	
	Q3: no	0	
	Q4: Domain-specific repository	2	
		8/17=**47%**	**F score**
	Q5: Publicly accessible	5	
	Q6: Non-standard web service	3	
	Q7: Unsure (data a. metadata are available but not clear if metadata will still be available when the data itself is removed)	0	
		8/10=**80%**	**A score**
	Q8: In a structured, open-standard, non-machine-readable format	1	
	Q9: Standardized vocabularies/ ontologies/ schema without global identifiers	2	
	Q10: The metadata record includes URI links to related metadata, data, and definitions	2	

		5/8=**63%**	**I score**
	Q11: Non-standard text-based license	2	
	Q12: Fully recorded in a text format	2	
		4/7=**57%**	**R score**
		47% x 0.25 + 80% x 0.25 + 63% x 0.25 + 57% x 0.25 =**62%**	**FAIR total score**
	O1: Publicly accessible but requires human intervention (e.g., may require login to download etc.)	**67 %**	**Openness score**

**Name of database**	**Answers to Q1-Q12 (ARDC)**	**Score**	**Criteria**
European Energy Efficiency Building District Database (Project: ExcEED)	Q1: Local identifier	1	
	Q2: Comprehensively, but in a text-based, non-standard format	3	
	Q3: No	0	
	Q4: The data is not described in any repository	0	
		4/17=**23%**	**F score**
	Q5: Unspecified conditional access e.g. contact the data custodian for access	2	
	Q6: By individual arrangement	1	
	Q7: No	0	
		3/10=**30%**	**A score**
	Q8: In a structured, open standard, non-machine-readable format	1	
	Q9: Standardized vocabularies/ontologies/schema without global identifiers -	2	
	Q10: There are no links to other metadata		
		3/8=**38%**	**I score**
	Q11: No license	0	
	Q12: No provenance information is recorded	0	
		0/7=**0%**	**R score**
		23% x 0.25 + 30% x 0.25 + 38% x 0.25 + 0% x 0.25 = **23%**	**FAIR total score**
	O1: Data are not open	0%	**Openness Score**

**Name of database**	**Answers to Q1-Q12 (ARDC)**	**Score**	**Criteria**
Global Buildings Performance Network (GBPN)	Q1: Globally unique, citable and persistent (e.g. DOI, PURL, ARK or Handle)	8	
	Q2: Brief title and description	2	
	Q3: No	0	
	Q4: Generalist public repository	2	
		12/17=**70%**	**F score**
	Q5: Publicly accessible	5	
	Q6: File download from online location	2	

	Q7: Unsure	0	
		7/10=**70%**	**A score**
	Q8: In a structured, open standard, non-machine-readable format	1	
	Q9: No standards have been applied in the description of data elements	1	
	Q10: The metadata record includes URI links to related metadata, data and definitions	2	
		4/8=**50%**	**I score**
	Q11: No license	0	
	Q12: Partially recorded	1	
		1/7=**14%**	**R score**
		70% x 0.25 + 70% x 0.25 + 50% x 0.25 + 14% x 0.25 = **51%**	**FAIR total score**
	O1: Publicly accessible but requires human intervention (e.g., may require login to download etc.)	2/3 = **67%**	**Openness Score**

**Name of database**	**Answers to Q1-Q12 (ARDC)**	**Score**	**Criteria**
The Open Source Mapping and Planning Tool for Cooling and Heating (Project: Hotmaps)	Q1: Web address (URL)	3	
	Q2: Comprehensively, but in a text-based, non-standard format	3	
	Q3: No	0	
	Q4: Local institutional repository	2	
		8/17=**47%**	**F score**
	Q5: Publicly accessible	5	
	Q6: File download from online location	2	
	Q7: Unsure	0	
		7/10=**70%**	**A score**
	Q8: In a structured, open standard, non-machine-readable format	2	
	Q9: Standardized vocabularies/ontologies/schema without global identifiers	2	
	Q10: There are no links to other metadata	0	
		4/8=**50%**	**I score**
	Q11: Standard machine-readable license (e.g. Creative Commons)	4	
	Q12: Fully recorded in a text format	2	
		6/7=**86%**	**R score**
		47% x 0.25 + 70% x 0.25 + 50% x 0.25 + 86% x 0.25 = **63%**	**FAIR total score**
	O1: Publicly accessible but requires human intervention (e.g., may require login to download etc.)	**67%**	**Openness score**

**Name of database**	**Answers to Q1-Q12 (ARDC)**	**Score**	**Criteria**
IEA Policy database	Q1: Web address (URL)	3	
	Q2: Comprehensively, but in a text-based, non-standard format	3	
	Q3: No	0	

	Q4: Local institutional repository	2	
		8/17=47**%**	**F score**
	Q5: Unspecified conditional access e.g. contact the data custodian for access	2	
	Q6: File download from online location	2	
	Q7: Yes	1	
		5/10=50**%**	**A score**
	Q8: In a structured, open-standard, non-machine-readable format	1	
	Q9: Standardized vocabularies/ontologies/schema without global identifiers	2	
	Q10: There are no links to other metadata	0	
		3/8=38**%**	**I score**
	Q11: Standard text-based license	3	
	Q12: Partially recorded	1	
		4/7=57**%**	**R score**
		47% x 0.25 + 50% x 0.25 + 38% x 0.25 + 57% x 0.25 = **48%**	**FAIR total score**
	O1: Publicly accessible by humans and machines alike by standard protocol (open, no log in etc. required)	**100%**	**Openness score**

**Name of database**	**Answers to Q1-Q12 (ARDC)**	**Score**	**Criteria**
IEA World Energy Statistics and Balances	Q1: Web address (URL)	3	
	Q2: Comprehensively, but in a text-based, non-standard format. Information spread across many files/websites.	3	
	Q3: No. Note, data needs to be purchased.	0	
	Q4: Local institutional repository.	2	
		8/17=47**%**	**F score**
	Q5: Unspecified conditional access e.g. contact the data custodian for access	2	
	Q6: File download from online location	2	
	Q7: Yes	1	
		5/10=50**%**	**A score**
	Q8: In a structured, open-standard, non-machine-readable format	1	
	Q9: Standardized vocabularies/ontologies/schema without global identifiers	2	
	Q10: To the extent open, there seems no links to other metadata	0	
		3/8=38**%**	**I score**
	Q11: Standard text-based license for purchasing the data.	3	
	Q12: Information not accessible (partially recorded is likely).	1	
		4/7=57**%**	**R score**
		47% x 0.25 + 50% x 0.25 + 38% x 0.25 + 57% x 0.25 = **48%**	**FAIR total score**
	O1: Data for purchase.	**0%**	**Openness score**

Name of database	Answers to Q1-Q12 (ARDC)	Score	Criteria
JRC data catalog	Q1: Web address (URL)	3	
	Q2: Brief title and description	2	
	Q3: Yes	1	
	Q4: Generalist public repository	2	
		8/17=47**%**	**F score**
	Q5: A de-identified / modified subset of the data is publicly accessible	4	
	Q6: File download from online location	2	
	Q7: Yes	1	
		7/10=70**%**	**A score**
	Q8: In a structured, open-standard, non-machine-readable format	1	
	Q9: Standardized vocabularies/ontologies/schema without global identifiers	2	
	Q10: The metadata record includes URI links to related metadata, data and definitions	2	
		5/8=63**%**	**I score**
	Q11: Standard text-based license	3	
	Q12: Partially recorded	1	
		5/7=57**%**	**R score**
		53% x 0.25 + 70% x 0.25 + 63% x 0.25 + 57% x 0.25 = **61%**	**FAIR total score**
	O1: Publicly accessible by humans and machines alike by standard protocol (open, no log in etc. required)	**100%**	**Openness score**

Name of database	Answers to Q1-Q12 (ARDC)	Score	Criteria
JRC data catalog (data collection)	Q1: Web address (URL)	3	
	Q2: Brief title and description	2	
	Q3: Yes	1	
	Q4: Generalist public repository	2	
		8/17=47**%**	**F score**
	Q5: Publicly accessible - the data set organization is visible to the users of the website showing the collection.	5	
	Q6: Standard web services (http) is used.	4	
	Q7: Yes	1	
		10/10=100**%**	**A score**
	Q8: Data (=names of the datasets available) are presented in a structured, open-standard, machine-readable format	2	
	Q9: No specific standards have been applied.	1	
	Q10: There are no links to other metadata	0	
		3/8=38**%**	**I score**
	Q11: Standard text-based license	3	
	Q12: Not systematically recorded, only mentions ‘updated irregularly’.	0	
		3/7=43**%**	**R score**

		47% x 0.25 + 100% x 0.25 + 38% x 0.25 + 43% x 0.25 = **57%**	**FAIR total score**
	O1: Publicly accessible by humans and machines alike by standard protocol (open, no log in etc. required)	**100%**	**Openness score**

Name of database	Answers to Q1-Q12 (ARDC)	Score	Criteria
JRC data catalogue (dataset)	Q1: Web address (URL)	3	
	Q2: Comprehensively, but in a text-based, non-standard format	3	
	Q3: No	0	
	Q4: Generalist institutional repository	2	
		8/17=47**%**	**F score**
	Q5: Public access when accepting the license.	5	
	Q6: File download from online location (with several redirections)	2	
	Q7: Yes	1	
		8/10=80**%**	**A score**
	Q8: In a structured, open-standard, non-machine-readable format	1	
	Q9: No standards have been applied.	1	
	Q10: There are no links to other metadata	0	
		2/8=25**%**	**I score**
	Q11: Standard text-based license	3	
	Q12: Not included in the metadata location. The zip-file from data download may contain such information, but is not accessible to humans without special visualization tools (e.g., using file-formats such as INF).	0	
		3/7=43**%**	**R score**
		47% x 0.25 + 80% x 0.25 + 25% x 0.25 + 43% x 0.25 = **49%**	**FAIR total score**
	O1: Human intervention required.	**66%**	**Openness score**

Name of database	Answers to Q1-Q12 (ARDC)	Score	Criteria
Mesures d'utilzation Rationelle d'Energi (MURE database)	Q1: Web address (URL)	3	
	Q2: Comprehensively, but in a text-based, non-standard format	3	
	Q3: No	0	
	Q4: Domain-specific repository	2	
		8/17=47**%**	**F score**
	Q5: Publicly accessible	5	
	Q6: File download from online location	2	
	Q7: Unsure	0	
		7/10=70**%**	**A score**
	Q8: In a structured, open-standard, non-machine-readable format	1	
	Q9: Standardized vocabularies/ontologies/schema without global identifiers	2	

	Q10: The metadata record includes URI links to related metadata, data and definitions	2	
		5/8=63**%**	**I score**
	Q11: No license	0	
	Q12: Fully recorded in a text format	2	
		2/7=29**%**	**R score**
		47% x 0.25 + 70% x 0.25 + 63% x 0.25 + 29% x 0.25 = **52%**	**FAIR total score**
	O1: Publicly accessible but requires human intervention (e.g., may require login to download etc.)	2/3 = **67%**	**Openness score**

**Name of database**	**Answers to Q1-Q12 (ARDC)**	**Score**	**Criteria**
Kaggle Repository	Q1: Yes, URL is assigned to metadata.	3	
	Q2: Very brief metadata description with title and description, only.	2	
	Q3: The dataset is not included in the files describing the data.	0	
	Q4: The data is published in a generalist public repository.	2	
		7/17=**41%**	**F score**
	Q5: Data is publicly accessible.	5	
	Q6: Data is available through download from an online location.	2	
	Q7: Yes, likely.	1	
		8/10=**80%**	**A score**
	Q8: The data are available as csv, open but not machine-readable as the headers are not explained.	1	
	Q9: No standards for vocabularies etc. have been used.	1	
	Q10: There are no links to other metadata.	0	
		2/8=**25%**	**I score**
	Q11: A standard text-based license is assigned.	3	
	Q12: Information about the updating frequency has not been provided.	0	
		3/7=**43%**	**R score**
		41% x 0.25 + 80% x 0.25 + 25% x 0.25 + 43% x 0.25 = **47%**	**FAIR total score**
	O1: Open for humans but not machines.	**66%**	**Openness score**

**Name of database**	**Answers to Q1-Q12 (ARDC)**	**Score**	**Criteria**
Simulations of hourily power output from wind and solar PV farms (renewables.ninja)	Q1: Globally unique, citable and persistent (e.g. DOI, PURL, ARK or Handle)	8	
	Q2: Comprehensively, but in a text-based, non-standard format	3	
	Q3: No	0	
	Q4: Domain-specific repository	2	
		13/17=**76%**	**F score**
	Q5: Publicly accessible	5	
	Q6: File download from online location	2	

	Q7: Unsure	0	
		7/10=**70%**	**A score**
	Q8: In a structured, open standard, machine-readable format	2	
	Q9: Standardized vocabularies/ontologies/schema without global identifiers	2	
	Q10: The metadata record includes URI links to related metadata, data and definitions	2	
		6/8=**75%**	**I score**
	Q11: Standard machine-readable license (e.g. Creative Commons)	4	
	Q12: Partially recorded	1	
		5/7=**71%**	**R score**
		76% x 0.25 + 70% x 0.25 + 75% x 0.25 + 71% x 0.25 = **73%**	**FAIR total score**
	O1: Publicly accessible but requires human intervention (e.g., may require login to download etc.)	**67%**	**Openness score**

**Name of database**	**Answers to Q1-Q12 (ARDC)**	**Score**	**Criteria**
U.S. National Renewable Energy Laboratory (NREL)	Q1: Globally unique, citable and persistent (e.g. DOI, PURL, ARK or Handle)	8	
	Q2: Comprehensively (see suggestion) using a recognized formal machine-readable metadata schema	4	
	Q3: Yes	1	
	Q4: Data is in one place but discoverable through several registries	4	
		17/17=100**%**	**F score**
	Q5: Publicly accessible	5	
	Q6: Standard web service API	4	
	Q7: Unsure	0	
		9/10=90**%**	**A score**
	Q8: In a structured, open-standard, machine-readable format	2	
	Q9: Standardized vocabularies/ontologies/schema without global identifiers	2	
	Q10: There are no links to other metadata	0	
		4/8=50**%**	**I score**
	Q11: No license	0	
	Q12: Fully recorded in a text format	2	
		2/7=29**%**	**R score**
		100% x 0.25 + 90% x 0.25 + 50% x 0.25 + 29% x 0.25 = **67%**	**FAIR total score**
	O1: Publicly accessible by humans and machines alike by standard protocol (open, no log in etc. required)	**100%**	**Openness score**

**Name of database**	**Answers to Q1-Q12 (ARDC)**	**Score**	**Criteria**
Scenarios of market	Q1: Web address (URL)	5	

transition to nearly zero energy buildings (NZEB)	Q2: Brief title and description	2	
	Q3: No	0	
	Q4: Generalist public repository	2	
		9/17=**53%**	**F score**
	Q5: Publicly accessible	5	
	Q6: By individual arrangement	1	
	Q7: Unsure	0	
		6/10=**60%**	**A score**
	Q8: In a structured, open standard, non-machine-readable format	1	
	Q9: Standardized vocabularies/ontologies/schema without global identifiers -	2	
	Q10: There are no links to other metadata	0	
		3/8=**38%**	**I score**
	Q11: No license	0	
	Q12: No provenance information is recorded	0	
		0/7=**0%**	**R score**
		53% x 0.25 + 60% x 0.25 + 38% x 0.25 + 0% x 0.25 = **38%**	**FAIR total score**
	O1: Publicly accessible but requires human intervention (e.g., may require login to download etc.)	2/3 = 67%	**Openness Score**

**Name of database**	**Answers to Q1-Q12 (ARDC)**	**Score**	**Criteria**
OECD hub (energy data)	Q1: Web address (URL)	3	
	Q2: Comprehensively (see suggestion) using a recognised, formal machine-readable metadata schema	4	
	Q3: yes	1	
	Q4: A)Data is in one place but discoverable through several registries	4	
		12/17=**70%**	**F score**
	Q5: Publicly accessible	5	
	Q6: : Standard web service API (e.g. OGC)	4	
	Q7: Unsure (data and metadata are available but not clear if metadata will still be available when the data itself is removed)	0	
		9/10=**90%**	**A score**
	Q8: I In a structured, open standard, machine-readable format	2	
	Q9: Standardized vocabularies/ontologies/schema without global identifiers	2	
	Q10: The metadata record includes URI links to related metadata, data, and definitions	2	
		6/8=**75%**	**I score**
	Q11: Standard machine-readable license (e.g. Creative Commons)	4	
	Q12: Fully recorded in a machine-readable format	3	

		7/7=**100%**	**R score**
		70% x 0.25 + 90% x 0.25 + 75% x 0.25 + 100% x 0.25 = **84%**	**FAIR total score**
	O1: Publicly accessible by humans and machines alike by standard protocol (open, no log in etc. required)	**100%**	**Openness score**

**Name of database**	**Answers to Q1-Q12 (ARDC)**	**Score**	**Criteria**
OECD - (data set)	Q1: Globally unique, citable, and persist	8	
	Q2: Comprehensive, but in a text-based, non-standard format	3	
	Q3: No	0	
	Q4: Local institutional repository	2	
		13/17=**77%**	**F score**
	Q5: Publicly accessible	5	
	Q6: File download from online location	2	
	Q7: Unsure	0	
		7/10=**70%**	**A score**
	Q8: In a structure, open standard, machine-readable format	2	
	Q9: No standards have been applied in the description of data elements.	1	
	Q10: There are no links to other metadata.	0	
		3/8=**38%**	**I score**
	Q11: Non-standard text-based license.	2	
	Q12: No provenance information is recorded.	0	
		2/7=**29%**	**R score**
		77% x 0.25 + 70% x 0.25 + 38% x 0.25 + 29% x 0.25 = **53%**	**FAIR total score**
	O1: Publicly accessible but requires human intervention (e.g., may require login to download etc.)	**67%**	**Openness score**

**Name of database**	**Answers to Q1-Q12 (ARDC)**	**Score**	**Criteria**
Heat Roadmap Europe (PETA)	Q1: Web address (URL)	5	
	Q2: Comprehensively, but in a text-based, non-standard format	3	
	Q3: No	0	
	Q4: Generalist public repository	2	
		10/17=**59%**	**F score**
	Q5: Publicly accessible	5	
	Q6: By individual arrangement	1	
	Q7: Unsure	0	
		6/10=**60%**	**A score**
	Q8: In a structured, open standard, non-machine-readable format	1	
	Q9: Standardized vocabularies/ontologies/schema without global identifiers -	2	
	Q10: There are no links to other metadata	0	

		3/8=**38%**	**I score**
	Q11: No license	0	
	Q12: No provenance information is recorded	0	
		0/7=**0%**	**R score**
		59% x 0.25 + 60% x 0.25 + 38% x 0.25 + 0% x 0.25 = **39%**	**FAIR total score**
	O1: Publicly accessible but requires human intervention (e.g., may require login to download etc.)	2/3 = **67%**	**Openness Score**

**Name of database**	**Answers to Q1-Q12 (ARDC)**	**Score**	**Criteria**
Database for support schemes, grid issues and policies about RE sources in EU-28, EFTA, etc. (RES LEGAL)	Q1: Web address (URL)	3	
	Q2: Comprehensively, but in a text-based, non-standard format	3	
	Q3: No	0	
	Q4: Local institutional repository	2	
		8/17=47**%**	**F score**
	Q5: Publicly accessible	5	
	Q6: File download from online location	2	
	Q7: Yes	1	
		8/10=80**%**	**A score**
	Q8: In a structured, open-standard, non-machine-readable format	1	
	Q9: Standardized vocabularies/ontologies/schema without global identifiers	2	
	Q10: There are no links to other metadata	0	
		3/8=38**%**	**I score**
	Q11: No license	0	
	Q12: Fully recorded in a text format	2	
		2/7=29**%**	**R score**
		47% x 0.25 + 80% x 0.25 + 38% x 0.25 + 29% x 0.25 = **49%**	**FAIR total score**
	O1: Publicly accessible by humans and machines alike by standard protocol (open, no log in etc. required)	**100%**	**Openness score**

**Name of database**	**Answers to Q1-Q12 (ARDC)**	**Score**	**Criteria**
Smart integration of energy storages in local multi-energy systems for maximizing the share of renewables in Europe's energy mix (Smiles)	Q1: Web address (URL)	3	
	Q2: Comprehensively (see suggestion) using a recognised, formal machine-readable metadata schema	4	
	Q3: No	0	
	Q4: Domain-specific repository	2	
		9/17=**53%**	**F score**
	Q5: Publicly accessible	5	
	Q6: File download from online location	2	
	Q7: Unsure	0	
		7/10=**70%**	**A score**
	Q8: In a structured, open standard, machine-readable format	2	

	Q9: Standardized vocabularies/ontologies/schema without global identifiers	2	
	Q10: There are no links to other metadata	0	
		4/8=**50%**	**I score**
	Q11: No license	0	
	Q12: Partially recorded	1	
		1/7=**14%**	**R score**
		53% x 0.25 + 70% x 0.25 + 50% x 0.25 + 14% x 0.25 = **47%**	**FAIR total score**
	O1: Publicly accessible but requires human intervention (e.g., may require login to download etc.)	**67%**	**Openness score**

**Name of database**	**Answers to Q1-Q12 (ARDC)**	**Score**	**Criteria**
EU Buildings Stock Monitoring (Project: EPISCOPE)	Q1: Web address (URL)	3	
	Q2: Comprehensively, but in a text-based, non-standard format	3	
	Q3: No	0	
	Q4: Domain-specific repository	2	
		8/17=**47%**	**F score**
	Q5: Publicly accessible	5	
	Q6: File download from online location	2	
	Q7: Unsure	0	
		7/10=**70%**	**A score**
	Q8: Mostly in a proprietary format	0	
	Q9: Standardized vocabularies/ontologies/schema without global identifiers -	2	
	Q10: There are no links to other metadata		
		2/8=**25%**	**I score**
	Q11: Non-standard text-based license	2	
	Q12: Partially recorded	1	
		3/7=**43%**	**R score**
		47% x 0.25 + 70% x 0.25 + 25% x 0.25 + 43% x 0.25 = **46%**	**FAIR total score**
	O1: Publicly accessible but requires human intervention (e.g., may require login to download etc.)	2/3=67%	**Openness Score**

**Name of database**	**Answers to Q1-Q12 (ARDC)**	**Score**	**Criteria**
Zenodo Repository (Data set 'Greco')	Q1: Globally unique, citable and persistent (e.g. DOI, PURL, ARK or Handle)	8	Web address (URL)
	Q2: Comprehensively (see suggestion) using a recognised, formal machine-readable metadata schema	4	Yes
	Q3: No	0	

	Q4: Data is in one place but discoverable through several registries	4	
		16/17=**94%**	**F score**
	Q5: Publicly accessible	5	Publicly accessible
	Q6: Standard web service API (e.g. OGC)	4	
	Q7: Unsure (data and metadata are available but not clear if metadata will still be available when the data itself is removed)	0	No
		9/10=**90%**	**A score**
	Q8: In a structured, open standard, machine-readable format	2	
	Q9: Standardized vocabularies/ontologies/schema without global identifiers	2	
	Q10: Metadata is represented in a machine readable format, e.g. in a linked data format such as Resource Description Framework (RDF)	3	
		7/8=**88%**	**I score**
	Q11: Standard machine-readable license (e.g. Creative Commons)	4	
	Q12: Fully recorded in a machine-readable format	3	
		7/7=**100%**	**R score**
		94% x 0.25 + 90% x 0.25 + 88% x 0.25 + 100% x 0.25 = **93%**	**FAIR total score**
	O1: Publicly accessible but requires human intervention (may require login to download etc.)	**100 %**	**Openness score**

**Name of database**	**Answers to Q1-Q12 (ARDC)**	**Score**	**Criteria**
Zenodo Repository (data set 'Sharewind')	Q1: Using a globally unique, persistent identifier.	8	
	Q2: Comprehensively, using a recognized formal machine-readable metadata schema (Open graph protocol).	4	
	Q3: The dataset is part of the metadata description.	1	
	Q4: Data is in one place (zenodo repository) but discoverable through several registries.	4	
		17/17=**100%**	**F score**
	Q5: Embargoed to ensure first author publication.	3	
	Q6: Standard web service API is used.	4	
	Q7: Yes, likely.	1	
		8/10=**80%**	**A score**
	Q8: Files are given as bmp-files, with limited access for algorithms.	1	
	Q9: Mixed - standardized but not systematically with global identifiers. Content itself is not a controlled vocabulary.	2	
	Q10: The metadata record includes URI links to related metadata, data, and definitions.	2	
		5/8=**63%**	**I score**
	Q11: Linked to a text-based license.	3	
	Q12: Partially recorded through versioning information.	1	
		4/7=**57%**	**R score**

		100% x 0.25 + 80% x 0.25 + 63% x 0.25 + 57% x 0.25 = **75%**	**FAIR total score**
	O1: Publicly available for machines and humans alike.	**100%**	**Openness score**

3.3 List of machine-based assessments of databases

Detailed results can be found in the Supplementary Material (spreadsheet), legend: *"Scores for calculating automated FAIR assessments in the low carbon energy domain"*, see Schwanitz & Wierling (2022) [10]**.** The tabular figure below illustrates a summary.

Fig. 3 Hotmap summarizing findings from machine-based FAIR assessments. F-1 to R-2 refer to FAIR maturity indicators in Wilkinson 2019 [9]. 80 databases have been assessed and sorted according to their compliance with the indicators. 17 points were the best performing databases. The test failed for 6 databases. Two databases were discontinued during the time when preparing this report.

F-1	F-2	F-3	F-4	F-5	F-6	F-7	F-8	A-1	A-2	A-3	A-4	A-5	I-1	I-2	I-3	I-4	I-5	I-6	I-7	R-1	R-2	SUM
1	0	1	0	0	1	0	1	1	0	1	1	1	1	1	1	1	1	1	1	1	1	17
1	0	1	0	0	1	0	1	1	0	1	1	1	1	1	1	1	1	1	1	1	1	17
1	0	1	0	0	1	0	1	1	0	1	1	1	0	0	1	1	1	1	1	1	1	15
1	0	1	0	0	1	0	1	1	0	1	1	1	0	0	1	1	1	1	1	1	1	15
1	0	1	0	0	1	0	1	1	0	1	1	1	0	0	1	1	1	1	1	1	1	15
1	0	1	0	0	1	0	1	1	0	1	1	1	0	0	1	1	1	1	1	1	1	15
1	0	1	0	0	1	0	1	1	0	1	1	1	0	1	1	1	1	1	1	0	0	14
1	0	1	0	1	1	0	1	1	0	1	1	1	0	0	1	1	1	1	1	0	0	14
1	0	1	0	0	1	0	1	1	0	1	1	1	0	0	1	1	1	0	0	1	1	13
1	0	1	0	0	1	0	1	1	0	1	1	1	0	0	1	1	1	1	1	0	0	13
1	0	1	0	0	1	0	1	1	0	1	1	1	0	0	1	1	1	1	1	0	0	13
1	0	1	0	0	1	0	1	1	0	1	0	1	0	1	1	1	1	1	1	0	0	13
1	0	1	0	0	1	0	1	1	0	1	1	1	0	0	1	1	1	1	1	0	0	13
0	0	1	0	0	1	0	1	0	0	1	0	1	0	0	1	1	1	1	1	1	1	12
0	0	1	1	1	1	0	1	0	0	1	0	1	0	0	1	1	1	1	1	0	0	12
0	0	1	1	0	1	0	1	0	0	1	0	1	0	0	1	1	1	1	1	0	0	11
0	0	1	1	0	1	0	1	0	0	1	0	1	0	0	1	1	1	1	1	0	0	11
0	0	1	0	0	1	0	1	0	0	1	0	1	0	0	1	1	1	0	0	1	1	10
0	0	1	0	0	1	0	1	0	0	1	0	1	0	0	1	1	1	1	1	0	0	10
0	0	1	0	0	1	0	1	0	0	1	0	1	0	0	1	1	1	1	1	0	0	10
0	0	1	0	0	1	0	1	0	0	1	0	1	0	0	1	1	0	0	0	1	1	9
0	0	1	0	0	1	0	1	0	0	1	0	1	0	0	1	1	1	0	0	0	0	8
0	0	1	0	0	1	0	1	0	0	1	0	1	0	0	1	1	1	0	0	0	0	8
0	0	1	0	0	1	0	1	0	0	1	0	1	0	0	1	1	1	0	0	0	0	8
0	0	1	0	0	1	0	1	0	0	1	0	1	0	0	1	1	1	0	0	0	0	8
0	0	1	0	0	1	0	1	0	0	1	0	1	0	0	1	1	1	0	0	0	0	8
0	0	1	0	0	1	0	1	0	0	1	0	1	0	0	1	1	0	0	1	0	0	8
0	0	1	0	0	1	0	1	0	0	1	0	1	0	0	1	1	1	0	0	0	0	8
0	0	1	0	0	1	0	1	0	0	1	0	1	0	0	1	1	0	0	0	0	0	7
0	0	1	0	0	1	0	1	0	0	1	0	1	0	0	1	1	0	0	0	0	0	7
0	0	1	0	0	1	0	1	0	0	1	0	1	0	0	1	1	0	0	0	0	0	7
0	0	1	0	0	1	0	1	0	0	1	0	1	0	0	1	1	0	0	0	0	0	7
0	0	1	0	0	1	0	1	0	0	1	0	1	0	0	1	1	0	0	0	0	0	7
0	0	1	0	0	1	0	1	0	0	1	0	1	0	0	1	1	0	0	0	0	0	7
0	0	1	0	0	1	0	1	0	0	1	0	1	0	0	1	1	0	0	0	0	0	7
0	0	1	0	0	1	0	1	0	0	1	0	1	0	0	1	1	0	0	0	0	0	7
0	0	1	0	0	1	0	1	0	0	1	0	1	0	0	1	1	0	0	0	0	0	7
0	0	1	0	0	1	0	1	0	0	1	0	1	0	0	1	1	0	0	0	0	0	7
0	0	1	0	0	1	0	1	0	0	1	0	1	0	0	1	1	0	0	0	0	0	7
0	0	1	0	0	1	0	1	0	0	1	0	1	0	0	1	1	0	0	0	0	0	7
0	0	1	0	0	1	0	1	0	0	1	0	1	0	0	1	1	0	0	0	0	0	7
0	0	1	0	0	1	0	1	0	0	1	0	1	0	0	1	1	0	0	0	0	0	7
0	0	1	0	0	1	0	1	0	0	1	0	1	0	0	1	1	0	0	0	0	0	7
0	0	1	0	0	1	0	1	0	0	1	0	1	0	0	1	1	0	0	0	0	0	7
0	0	1	0	0	1	0	1	0	0	1	0	1	0	0	1	1	0	0	0	0	0	7
0	0	1	0	0	1	0	1	0	0	1	0	1	0	0	1	1	0	0	0	0	0	7
0	0	1	0	0	1	0	1	0	0	1	0	1	0	0	1	1	0	0	0	0	0	7
0	0	1	0	0	1	0	1	0	0	1	0	1	0	0	1	1	0	0	0	0	0	7
0	0	1	0	0	1	0	1	0	0	1	0	1	0	0	1	1	0	0	0	0	0	7
0	0	1	0	0	1	0	1	0	0	1	0	1	0	0	1	1	0	0	0	0	0	7
0	0	1	0	0	1	0	1	0	0	1	0	1	0	0	1	1	0	0	0	0	0	7
0	0	1	0	0	1	0	1	0	0	1	0	1	0	0	1	1	0	0	0	0	0	7
0	0	1	0	0	1	0	1	0	0	1	0	1	0	0	1	1	0	0	0	0	0	7
0	0	1	0	0	1	0	1	0	0	1	0	1	0	0	1	1	0	0	0	0	0	7
0	0	0	0	0	0	0	1	0	0	1	0	1	0	0	0	0	0	0	0	0	0	3
0	0	0	0	0	0	0	1	0	0	1	0	1	0	0	0	0	0	0	0	0	0	3
0	0	0	0	0	0	0	1	0	0	1	0	1	0	0	0	0	0	0	0	0	0	3
0	0	0	0	0	0	0	1	0	0	1	0	1	0	0	0	0	0	0	0	0	0	3
0	0	0	0	0	0	0	1	0	0	1	0	1	0	0	0	0	0	0	0	0	0	3
0	0	0	0	0	0	0	1	0	0	1	0	1	0	0	0	0	0	0	0	0	0	3
0	0	0	0	0	0	0	1	0	0	1	0	1	0	0	0	0	0	0	0	0	0	3
0	0	0	0	0	0	0	1	0	0	1	0	1	0	0	0	0	0	0	0	0	0	3
0	0	0	0	0	0	0	1	0	0	1	0	1	0	0	0	0	0	0	0	0	0	3
0	0	0	0	0	0	0	1	0	0	1	0	1	0	0	0	0	0	0	0	0	0	3
0	0	0	0	0	0	0	1	0	0	1	0	1	0	0	0	0	0	0	0	0	0	3
0	0	0	0	0	0	0	1	0	0	1	0	1	0	0	0	0	0	0	0	0	0	3
0	0	0	0	0	0	0	1	0	0	1	0	1	0	0	0	0	0	0	0	0	0	3
0	0	0	0	0	0	0	1	0	0	1	0	1	0	0	0	0	0	0	0	0	0	3
0	0	0	0	0	0	0	1	0	0	1	0	1	0	0	0	0	0	0	0	0	0	3
0	0	0	0	0	0	0	1	0	0	1	0	1	0	0	0	0	0	0	0	0	0	3
0	0	0	0	0	0	0	1	0	0	1	0	1	0	0	0	0	0	0	0	0	0	3
0	0	0	0	0	0	0	1	0	0	1	0	1	0	0	0	0	0	0	0	0	0	3
																						0
																						0
																						0
																						0
																						0
																						0
																						0
																						0
13	0	54	3	2	54	0	72	13	0	72	12	72	2	4	54	54	26	18	19	10	10	

References

1. Global Energy Assessment Writing Team. *Global Energy Assessment - Toward a Sustainable Future.* International Institute for Applied Systems Analysis, Vienna, Austria and Cambridge University Press, Cambridge, UK and New York, NY, USA. (2012). ISBN: 9780521182935.

2. Standard International Energy Product Classification (SIEC). Available at: https://unstats.un.org/unsd/classifications/Family/Detail/2007 (2011).

3. Global Change Master Directory *Keywords.* NASA. Available at: https://earthdata.nasa.gov/earth-observation-data/find-data/gcmd/gcmd-keywords (2020).

4. American Economic Association. JEL Classification System / EconLit Subject Descriptors. Available at: https://www.aeaweb.org/econlit/jelCodes.php?view=jel (2022).

5. The European Science Vocabulary (EuroSciVoc). Available at: https://op.europa.eu/en/web/eu-vocabularies/euroscivoc (2020).

6. Wilkinson, M.D. *et al.* The FAIR Guiding Principles for scientific data management and stewardship. *Sci Data* **3**, 160018 (2016). DOI: https://doi.org/10.1038/sdata.2016.18.

7. Bahim, C., Dekkers, M., *&* Wyns B. Results of an Analysis of Existing FAIR assessment tools. Research Data Alliance. (2019) DOI: https://doi.org/10.15497/RDA00035.

8. Australian Research Data Commons (ARDC). *FAIR self assessment tool* (2020) https://ardc.edu.au/resources/working-with-data/fair-data/fair-self-assessment-tool/ (Note also: The code for this tool is available for reuse on Github.

9. Wilkinson, M.D. *et al.* Evaluating FAIR maturity through a scalable, automated, community-governed framework. *Sci Data* **6**, 174 (2019). DOI: https://doi.org/10.1038/s41597-019-0.

10. Schwanitz, V.J. & Wierling, A. Scores for calculating automated FAIR assessments in the low carbon energy domain. Zenodo. Available at: https://zenodo.org/record/5577964#.YiIwM3VKjeQ (2022).
